# Smoking, education level and excess premature deaths in the USA

**DOI:** 10.1038/s44360-026-00153-7

**Published:** 2026-07-10

**Authors:** Xuyang Tang, Eo Rin Cho, Patrick Brown, Kenneth E. Warner, Prabhat Jha

**Affiliations:** 1 Centre for Global Health Research, Dalla Lana School of Public Health, Toronto, Ontario Canada; 2 Oxford Population Health, Nuffield Department of Population Health, University of Oxford, Oxford, UK; 3 Department of Statistical Sciences, University of Toronto, Toronto, Ontario Canada; 4 School of Public Health, University of Michigan, Ann Arbor, MI USA

**Keywords:** Epidemiology, Risk factors

## Abstract

Adult survival among non-Hispanic white (‘white’) people in the USA has stagnated in recent decades, driven by rising mortality among adults with low educational attainment. We examined national mortality and population data to quantify the contributions of smoking-attributable diseases and selected other causes—notably opioids—to survival between ages 30 and 79 years from 1992 to 2019, stratified by three education levels: ≤11 years (low), 12 years (middle) and ≥13 years (high). We found that the absolute gap in the 50-year risk of death between low- and high-education white adults doubled among men and tripled among women. Smoking-attributable mortality increased markedly among low-education white adults but decreased in the two higher-education groups. Among 3.2 million excess premature deaths in low- and middle-education adults, smoking accounted for 63%. At ages 30–64 years, opioids accounted for 3% of excess deaths, rising to 8% during 2010–2019. Smoking-attributable mortality is the leading contributor to stagnating survival among white adults in the USA.

## Abstract (web summary)

Analysis of US mortality data from 1992 to 2019 shows widening educational inequalities in survival among non-Hispanic white adults, with smoking accounting for nearly two-thirds of excess premature deaths in lower-educated groups and outweighing the contributions of opioids.

## Main

A marked stagnation in adult survival in the USA has emerged in recent decades^1,2^, driven largely by rising mortality rates among less educated individuals^3,4^. Non-Hispanic white (hereafter ‘white’) populations—comprising approximately 80% of the US population in 1990 and 60% in 2020 (ref. ^5^)—exhibit the greatest stagnation in survival. By contrast, the survival of US Black adults has improved, and the survival patterns of those with Hispanic ethnicity in the USA are strongly influenced by the in-migration of healthier cohorts^2–4^. The pattern of stagnation in survival among US white adults emerged alongside deindustrialization and sluggish wage growth among low- and middle-income workers^6–10^, paired with more recent sharp increases in opioid overdoses and related deaths^11^. These trends were popularized as the ‘diseases of despair’, a term coined by Case and Deaton^12^ to describe rising mortality from opioids, accidental poisoning, suicide, cirrhosis and other alcohol-related causes.

Subsequent analyses have shown that stagnation in survival also reflects widening educational disparities in mortality from cancer, cardiovascular disease and chronic obstructive pulmonary disease (COPD)^13,14^. However, the contribution of smoking to the marked divergence in survival across educational groups has received comparatively little attention, despite smoking accounting for at least one in seven adult deaths in 2020. Although smoking prevalence has declined in all education groups, the decline has been substantially slower among individuals with lower education and among those aged 50 years or older^15–17^.

We hypothesize that smoking-attributable mortality is the major contributor to the widening educational gradients in mortality among the US white population. Prevalence data alone cannot reliably predict subsequent cause-specific mortality from smoking, and sufficiently large prospective studies to quantify long-term educational gradients in smoking-attributable mortality do not yet exist. Thus, to test our hypothesis, we use the method developed by Peto et al.^18^, which treats observed lung cancer mortality as an index of cumulative smoking exposure. We then combine this calculated exposure with smoker–nonsmoker relative risks (RRs) for other diseases. We apply this method to three groups based on years of education: ≤11 years (low-education group), 12 years (middle-education group) and ≥13 years (high-education group). A key strength of this method is that lung cancer mortality captures age, period and cohort effects—including variation in cigarette type, age at initiation, duration of smoking and cessation—without complex modelling of these factors. Although crude, the method minimizes the complexities inherent in disentangling cohort and period effects^19^.

We assess mortality over a 30-year period, decompose deaths into smoking-attributable and non-smoking-attributable components, and examine educational gradients in both midlife and older ages. We separately quantify the trends in mortality from opioids, accidental poisoning, cirrhosis, suicide, injuries and other external causes. Finally, we estimate excess deaths attributable to smoking and other causes in the two lower-education groups relative to the highest-education group. We further examine possible biases in the method and find that, even when accounting for such biases, the lung cancer-based index method robustly documents the sharp divergence in smoking-attributable mortality in lower-education groups. Our findings underscore the urgent need for public health action, particularly expanded smoking cessation efforts, to reduce the extraordinarily high mortality rates among adults with lower levels of education.

## Results

From 1992 to 2024, 33 million deaths among US white adults with known education status occurred at ages 30–79 years, of which 19 million occurred in men and 14 million in women; 13 million occurred at ages 30–64 and 20 million at ages 65–79. Relative to 1992, the high-education population among white adults aged 30–79 years increased by 60% in men and 88% in women by 2024, in contrast to declines of 68% and 75%, respectively, in the low-education population.

**Fig. 1:**
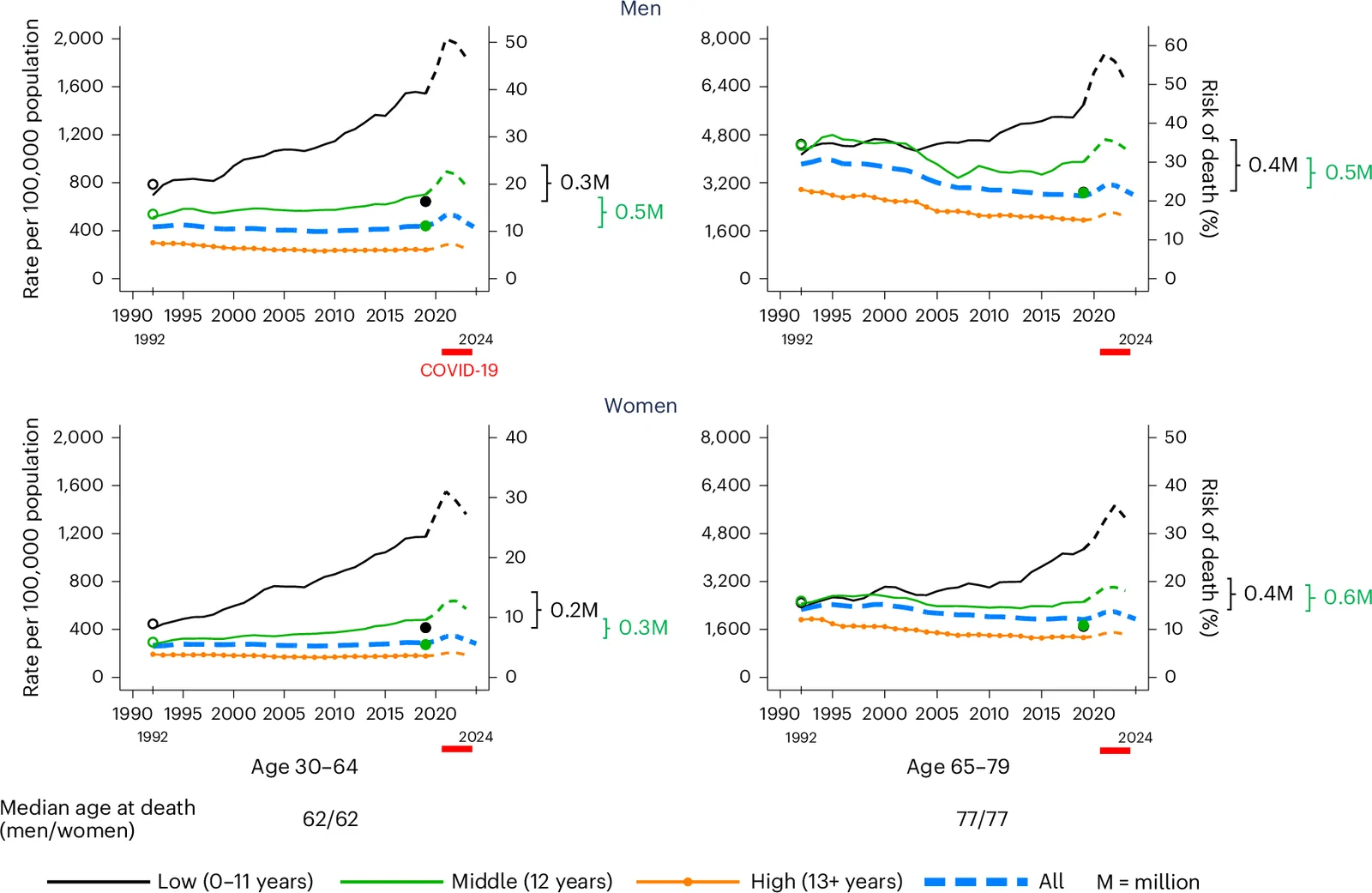
Two-year moving average of age-standardized all-cause mortality rates in white adults by age group and years of education, 1992–2024. Mortality rates are adjusted to the 2000 US standard population. White refers to non-Hispanic white individuals. Mortality affected by the COVID-19 pandemic is shown with a dotted line. The *y*-axis scales for the age group 65–79 are four times those of the age group 30–64. Of 64,174,696 deaths, 12% occurred in men and 8% in women aged 30–64 years; 18% in men and 14% in women aged 65–79 years; and 19% in men and 28% in women aged 80 years or older. The median age at death is shown below the *x* axes. For men aged 30–64 with 0–11, 12 and 13+ years of education, the AARRs are −3%, −1% and −1% (that is, a 1% decrease in mortality). For women in the same age group, the AARRs are −4%, −2% and 0.3%. For men aged 65–79, the AARRs are −1%, 1% and 2%. For women, the AARRs are −2%, 0.31% and −1%. Open circles denote the starting year of the AARR calculation and closed circles denote the ending year.

Among low-education white men, the 50-year risk of death between ages 30 and 79 years increased from 65% in 1992–1995 to 79% in 2016–2019 (Table 1). A comparable increase was observed in women, from 44% to 67%. Among middle-education men, the risk of death declined modestly from 62% to 60%; among women, it rose modestly from 42% to 44%. By contrast, in the high-education group, the risk of death declined from 44% to 34% in men and from 31% to 25% in women. As a result, over the three decades analysed, the absolute gap in the risk of death between low- and high-education men more than doubled, from 21 to 44 percentage points, and the gap among women tripled, from 13 to 42 percentage points. The gap in the risk of death between the middle- and high-education groups increased by about 50% in men and doubled in women. The COVID-19 pandemic accentuated these disparities, with the risk of death at ages 30–79 years rising 8–10% more in the two lower-education groups, until declines began in all education groups in 2022 (Fig. 1). The subsequent analyses of long-term stagnation in survival focus on the pre-COVID-19 period, specifically from 1992 to 2019.

**Table 1:** Five- and thirty-year averages of population, deaths, age-adjusted mortality rates and 50-year risk of death by year, education and sex in white adults aged 30–79 years, 1992–2019

Period	Population/deaths (millions)			Rate per 100,000 population			Risk of death at ages 30–79 years			Absolute gap in risks	
	Low education (0–11 years)	Middle education (12 years)	High education (13+ years)	Low education	Middle education	High education	Low education	Middle education	High education	Between middle and high education	Between low and high education
**Men**											
**Annual average over the comparison period**											
1992–1995	7/0.2	18/0.2	27/0.2	1,500	1,293	751	65%	62%	44%	18%	21%
2016–2019	3/0.1	17/0.3	39/0.3	2,377	1,291	558	79%	60%	34%	25%	44%
2021–2024	2/0.1	17/0.3	40/0.3	2,871	1,483	593	84%	64%	36%	28%	48%
**Annual average over 28 years (ignoring COVID-19 effects)**											
1992–2019	5/0.1	18/0.2	33/0.2	1,836	1,230	633	70%	59%	38%	21%	32%
**Women**											
**Annual average over the comparison period**											
1992–1995	7/0.1	22/0.2	25/0.1	823	700	473	44%	42%	31%	10%	13%
2016–2019	3/0.1	17/0.2	42/0.2	1,680	821	377	67%	44%	25%	19%	42%
2021–2024	2/0.1	16/0.2	44/0.2	2,021	987	403	74%	50%	26%	23%	47%
**Annual average over 28 years (ignoring COVID-19 effects)**											
1992–2019	5/0.1	20/0.2	34/0.1	1,179	734	408	54%	42%	27%	15%	27%

Mortality rates are adjusted to the 2000 US standard population. The 50-year smoking-attributable risk of death is defined as 1 − exp(−∑_*p*_ 50*R*_*p*_/100,000), where *R*_*p*_ is the 5-year age-specific all-cause mortality rate for the ten 5-year periods (30–34, 35–39 and so on to 75–79 years). White refers to non-Hispanic white individuals. In 2021, the 50-year risk of death for adults aged 30–79 years was 31% in Canada and 74% in low-income countries, calculated using population and death estimates from the UN Population Division.

### Mortality trends by age and education

**Extended Data Fig. 1:**
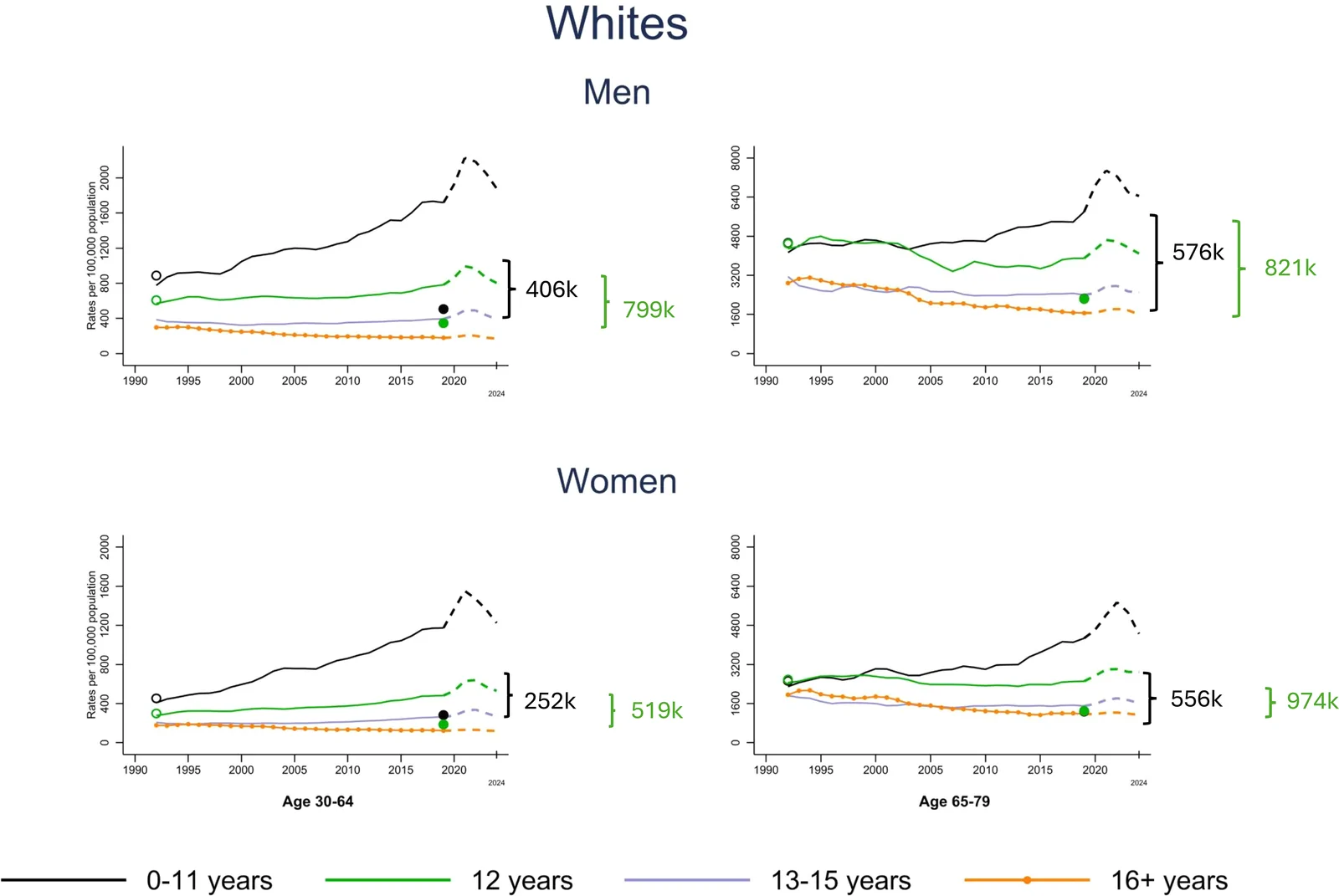
Sensitivity analysis on re-categorizing education to 4 levels (0-11, 12, 13-15, 16+ years) in whites by age groups, 1992-2024. Notes: Mortality rates adjusted to 2000 US standard population. White refer to Non-Hispanic white individuals. Mortality affected by COVID-19 pandemic in dotted line. Y-axis scales for age group 65-79 are 4 times that of age group 30-64.

For the entire population, the age-standardized all-cause mortality rates declined modestly from 1992 to 2019 at ages 30–64 but much more steeply at ages 65–79. In the high-education group, death rates declined steadily across both age groups and in both sexes. By contrast, mortality rates in the low- and middle-education groups diverged substantially from each other and from the high-education group. In the lower-education groups, 54% of men and 40% of women are expected to die between the ages of 30 and 64, based on the death rates observed in 2019 (secondary *y* axis in Fig. 1). In 1992, the risk of death among those with low education was 28% for men and 16% for women. At ages 65–79, mortality rates were similar between the low- and middle-education groups until around 2005, after which they diverged as mortality rates rose in the low-education group. We also evaluated individuals who completed college (16 or more years of education) as the reference group. The results were consistent with those obtained for adults with 13 or more years of education (Extended Data Fig. 1) but yielded substantially larger estimates of excess mortality (see ‘Excess deaths in the two lower-education groups’ and Extended Data Fig. 1 for more details) in the lower-education groups. There was variability among states in achieving high coverage of education reporting on death certificates. However, our sensitivity analyses showed little difference when states with low completeness of education data were excluded (Methods).

### Excess deaths in the two lower-education groups

To estimate excess deaths in the two lower-education groups, we first constructed a counterfactual scenario in which mortality rates in these groups were presumed to decline from 1992 to 2019 at the same annual rate of reduction observed in the highest-education group. We then compared these counterfactual mortality rates (shown as black and green circles for the low- and middle-education groups, respectively, in Fig. 1) with the observed mortality rates from 1992 to 2019. The difference between observed and counterfactual mortality yields an estimate of cumulative excess deaths, defined as deaths that would not have occurred had mortality rates in the two lower-education groups mirrored those in the highest-education group. This does not assume, however, convergence of absolute mortality rates, which are highest in the two lower-education groups. Over the study period, the observed annual mortality rate increased by 3% among men and 4% among women in the low-education group, whereas it declined by 1% among men and 0.3% among women in the high-education group (Fig. 1).

Applying the counterfactual framework yielded an estimated 3.2 million cumulative excess deaths between 1992 and 2019 in the two lower-education groups. Of these excess deaths, 1.3 million occurred among individuals aged 30–64, including 0.3 million men and 0.2 million women in the low-education group, and 0.5 million men and 0.3 million women in the middle-education group. An additional 1.9 million excess deaths occurred at ages 65–79, comprising 0.4 million men and 0.4 million women in the low-education group, and 0.5 million men and 0.6 million women in the middle-education group.

### Trends in smoking-attributable mortality and other causes of death

**Fig. 2:**
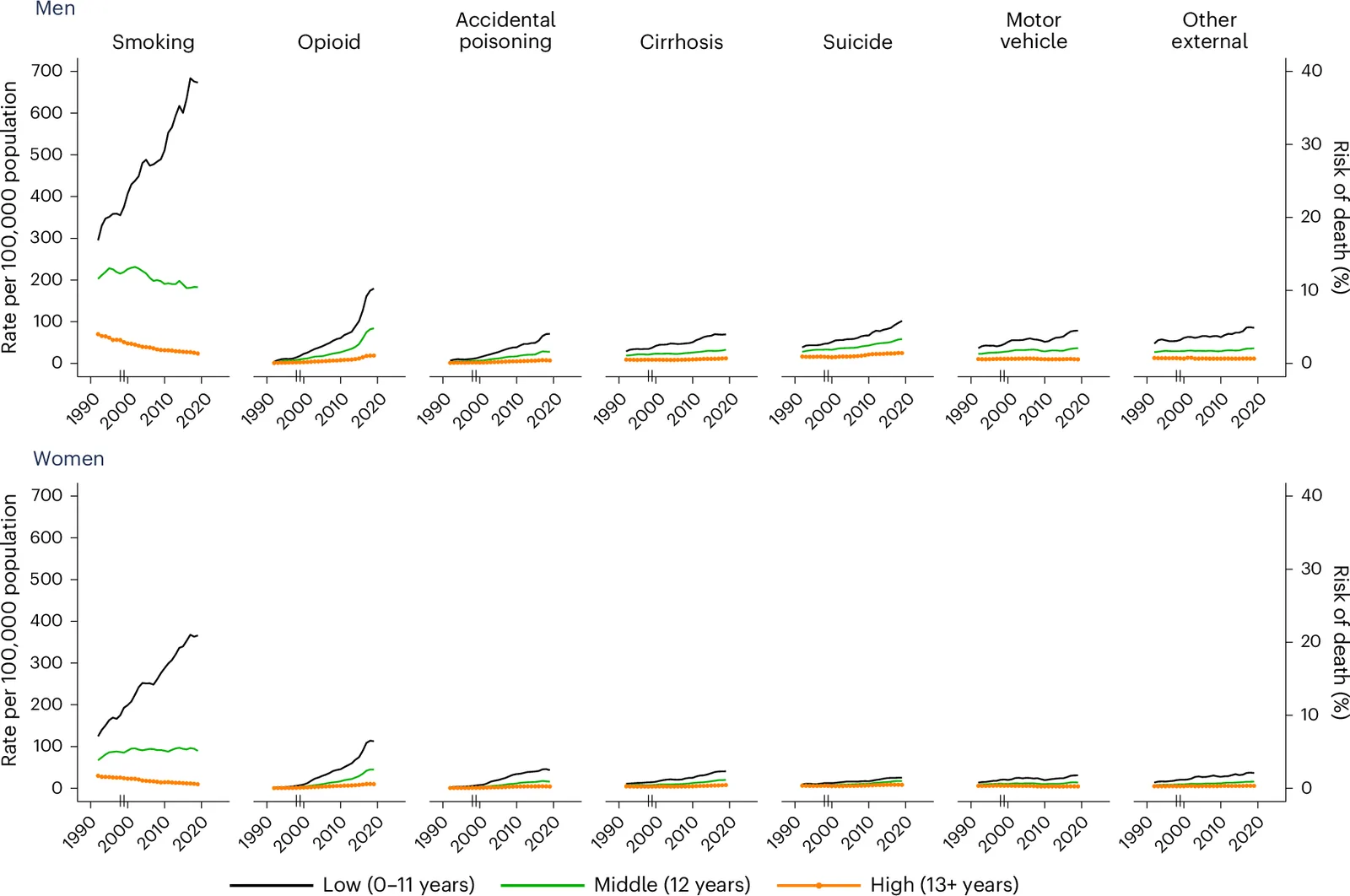
Two-year moving average of cause-specific age-adjusted mortality rates for selected causes in white men and women aged 30–64 by years of education, 1992–2019. Mortality rates are adjusted to the 2000 US standard population. White refers to non-Hispanic white individuals. Double bars on the *x* axis reflect the change from ICD-9 to ICD-10 in 1999.

**Extended Data Fig. 2:**
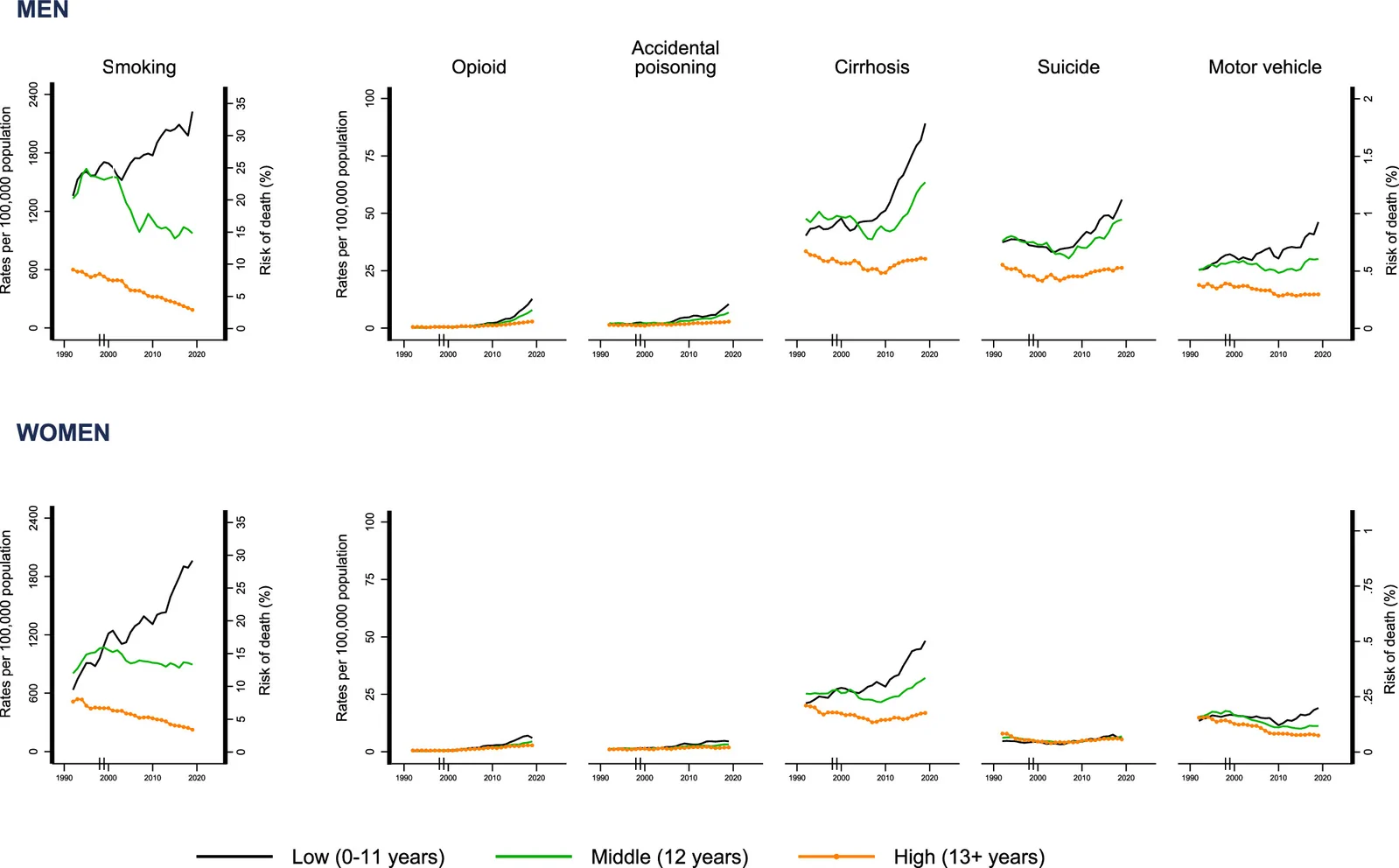
Two-year moving average of cause-specific age-adjusted mortality rates for selected causes in white men and women aged 65-79 by years of education, 1992-2019. Notes: Mortality rates adjusted to 2000 US standard population. White refers to Non-Hispanic white individuals. Double bars on the x-axis reflect the change from ICD-9 to ICD-10 in 1999. Y-axis scales for Smoking are 24 times the scale of all other conditions.

Age-adjusted smoking-attributable death rates increased in the low-education group at ages 30–64 years (Fig. 2) and 65–79 years (Extended Data Fig. 2), whereas they declined in the middle- and high-education groups. Declines in smoking-attributable death rates in the middle- and high-education groups were steeper in men than in women, reflecting earlier patterns of quitting among men. At ages 30–64, mortality rates in the two lower-education groups increased due to opioids, accidental poisoning, cirrhosis, suicide, motor vehicle accidents and other external causes. The sharpest increase in opioid-related mortality occurred after 2010. Increases were larger in men than in women, particularly for cirrhosis, suicide and motor vehicle accidents. Death rates from these causes also rose in the high-education group, with the exception of motor vehicle accidents and other external causes.

At ages 65–79, smoking-attributable deaths far outnumbered deaths from other causes (Extended Data Fig. 2 presents these deaths on a vertical scale 20 times larger than that for other causes). At these ages, mortality rates increased for cirrhosis and accidental falls in both sexes and for suicide and motor vehicle accidents in men. At ages 65–79, the rates of opioid- and accidental poisoning-related deaths did not increase, except for small increases in the two lower-education groups after approximately 2015. Supplementary Table 2 provides the International Classification of Diseases (ICD) codes used for these diagnoses and for smoking-attributable diseases.

**Fig. 3:**
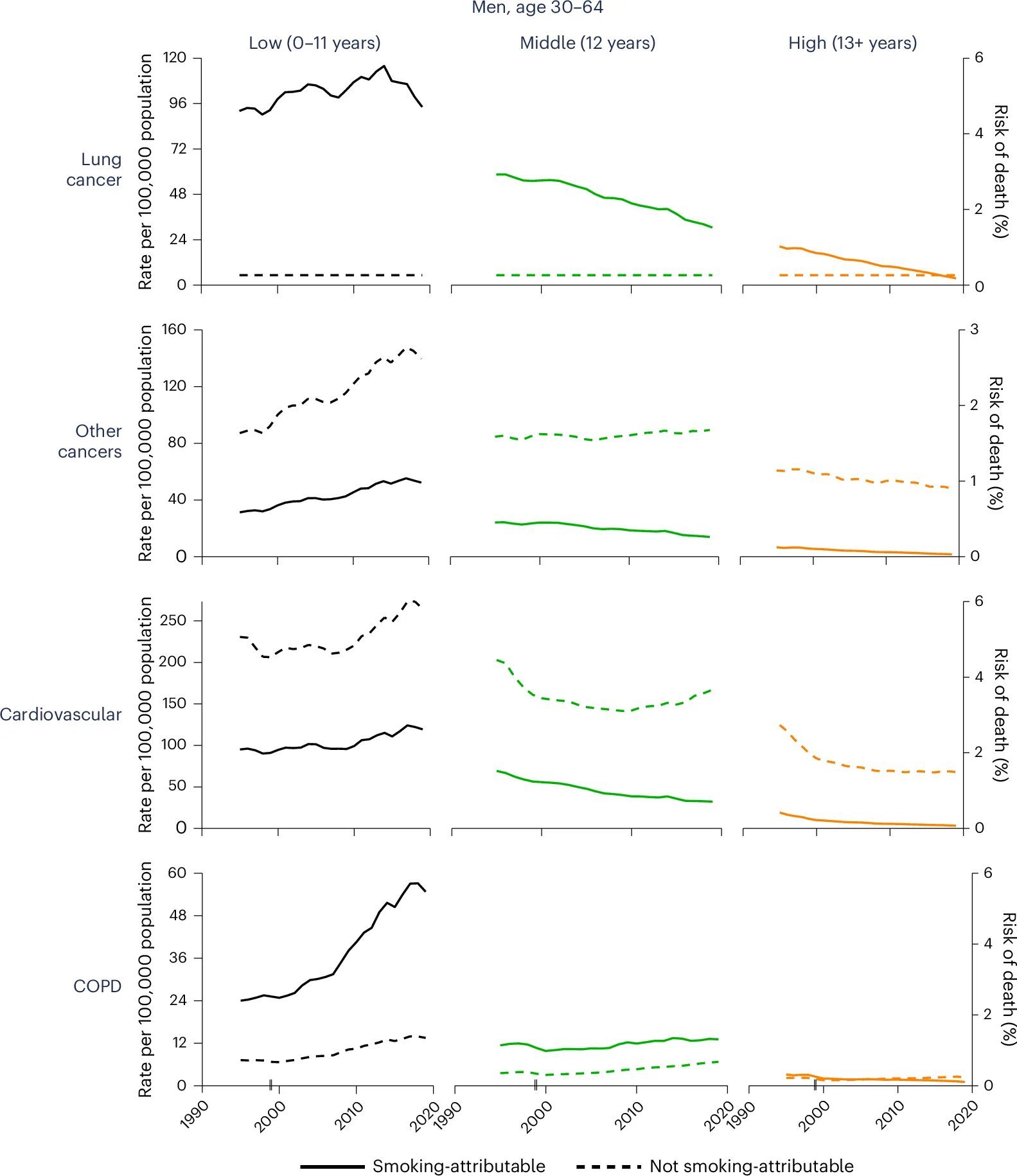
Two-year moving average of cause-specific age-adjusted mortality rates for selected smoking-related causes in white men aged 30–64 by years of education, 1992–2019. Mortality rates are adjusted to the 2000 US standard population. White refers to non-Hispanic white individuals. Double bars on the *x* axis reflect the change from ICD-9 to ICD-10 in 1999.

**Fig. 4:**
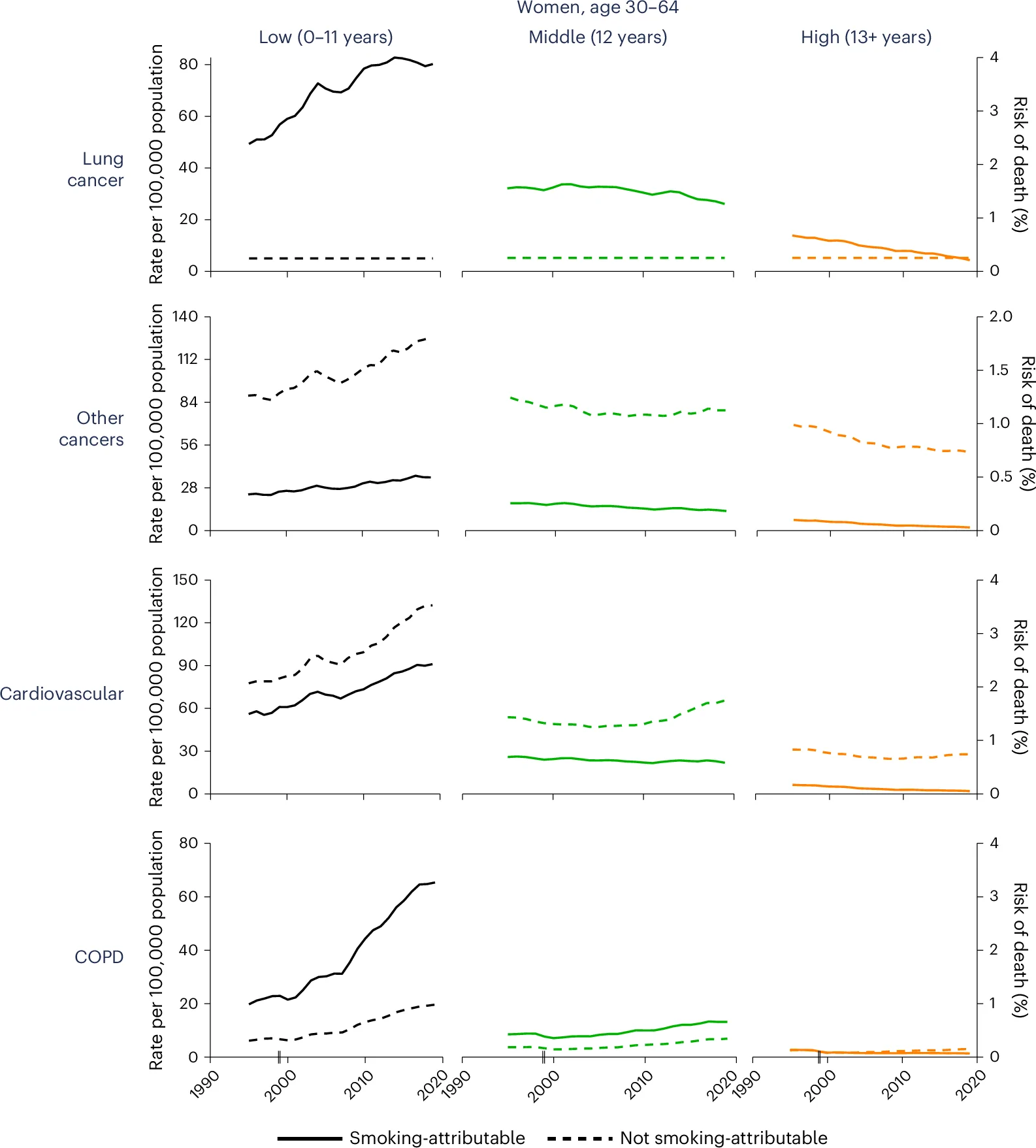
Two-year moving average of cause-specific age-adjusted mortality rates for selected smoking-related causes in white women aged 30–64 by years of education, 1992–2019. Mortality rates are adjusted to the 2000 US standard population. White refers to non-Hispanic white individuals. Double bars on the *x* axis reflect the change from ICD-9 to ICD-10 in 1999.

**Extended Data Fig. 3:**
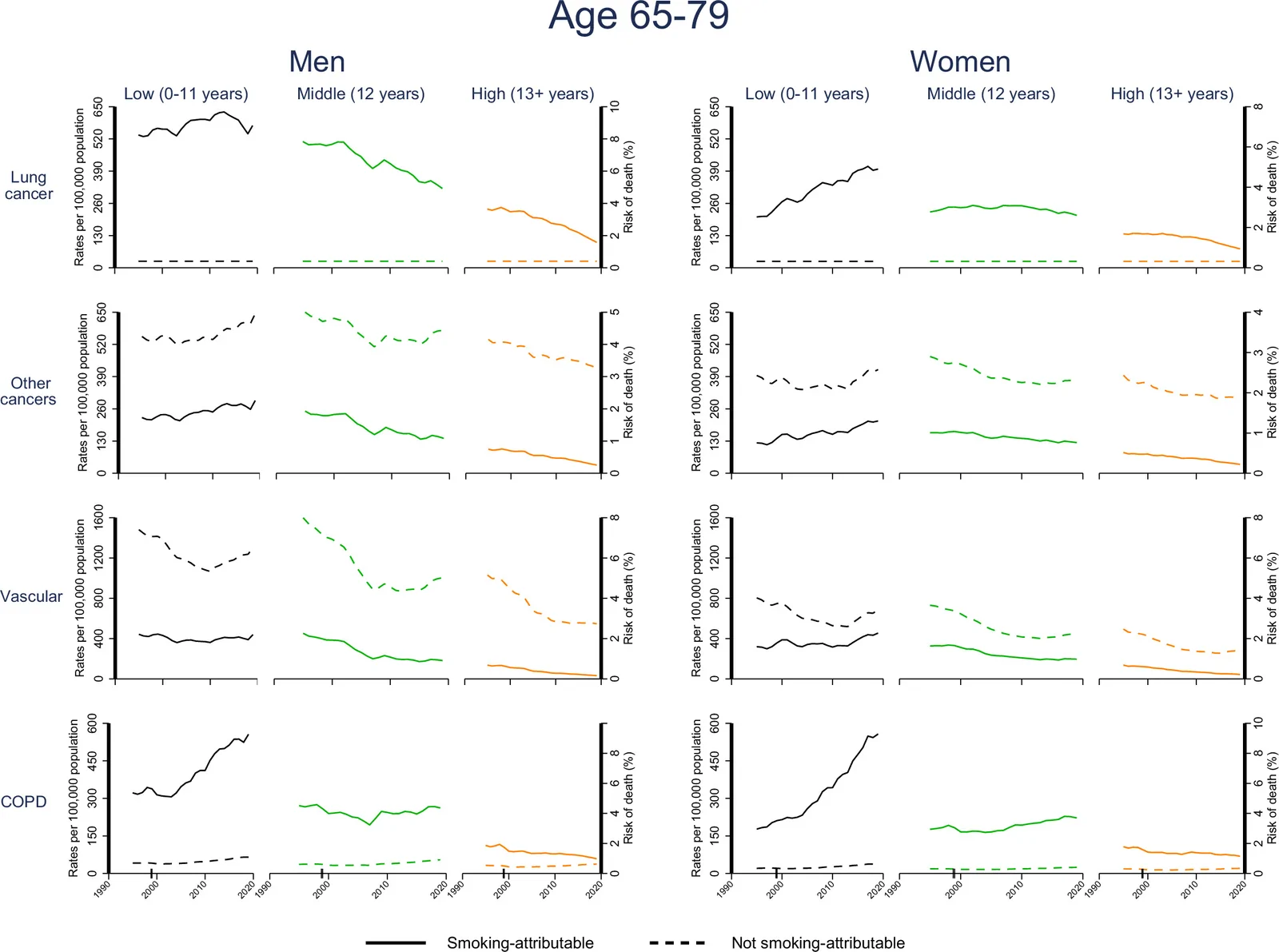
Two-year moving average of smoking-related cause-specific age-adjusted mortality rates for selected causes in white men and women aged 65-79 by years of education, 1992-2019. Notes: Mortality rates adjusted to 2000 US standard population. White refers to Non-Hispanic white individuals. Plots for men and women do not share the same secondary Y-axis scale (that is Risks). Double bars on the x-axis reflect the change from ICD-9 to ICD-10 in 1999.

The indirect method allows lung cancer, other cancers, cardiovascular disease and COPD to be divided into fractions attributable to smoking and those not attributable to smoking. We applied this procedure for each education stratum and sex at ages 30–64 years (Figs. 3 and 4) and 65–79 years (Extended Data Fig. 3). In low-education men aged 30–64, smoking-attributable mortality rose for lung cancer, other cancers, cardiovascular disease and COPD, with a sharp increase noted in COPD mortality after 2010, followed by a modest decline starting around 2015. Smoking-attributable lung cancer mortality rose until 2015 and declined somewhat thereafter. In low-education men, mortality rates not attributable to smoking also increased, particularly for other cancers and cardiovascular disease.

By contrast, among middle-education men at these ages, smoking-attributable mortality rates for lung cancer, other cancers and cardiovascular disease declined, while they remained flat for COPD. Mortality rates not attributable to smoking rose modestly for other cancers and COPD but declined initially and then rose from 2010 for cardiovascular disease. In highly educated men, both mortality attributable to smoking and mortality not attributable to smoking generally declined, and absolute rates were substantially lower than those in men with middle or low education.

Women aged 30–64 of various education levels showed similar educational patterns to men but generally had lower mortality risks. Low-education women also showed a sharp increase in lung cancer and COPD mortality attributable to smoking, with some stabilization around 2015. Cardiovascular mortality, both attributable and not attributable to smoking, rose notably among women with low education. Cardiovascular mortality not attributable to smoking increased somewhat among middle-education women and remained flat among high-education women. Although the overall absolute increase in age-adjusted smoking-attributable death rates was larger in men, the relative trends were comparable between the sexes. Broadly similar patterns for each sex were also observed at ages 65–79 (Extended Data Fig. 3), with sharp increases in COPD mortality attributable to smoking and a plateau in declines in cardiovascular mortality not attributable to smoking.

**Extended Data Fig. 4:**
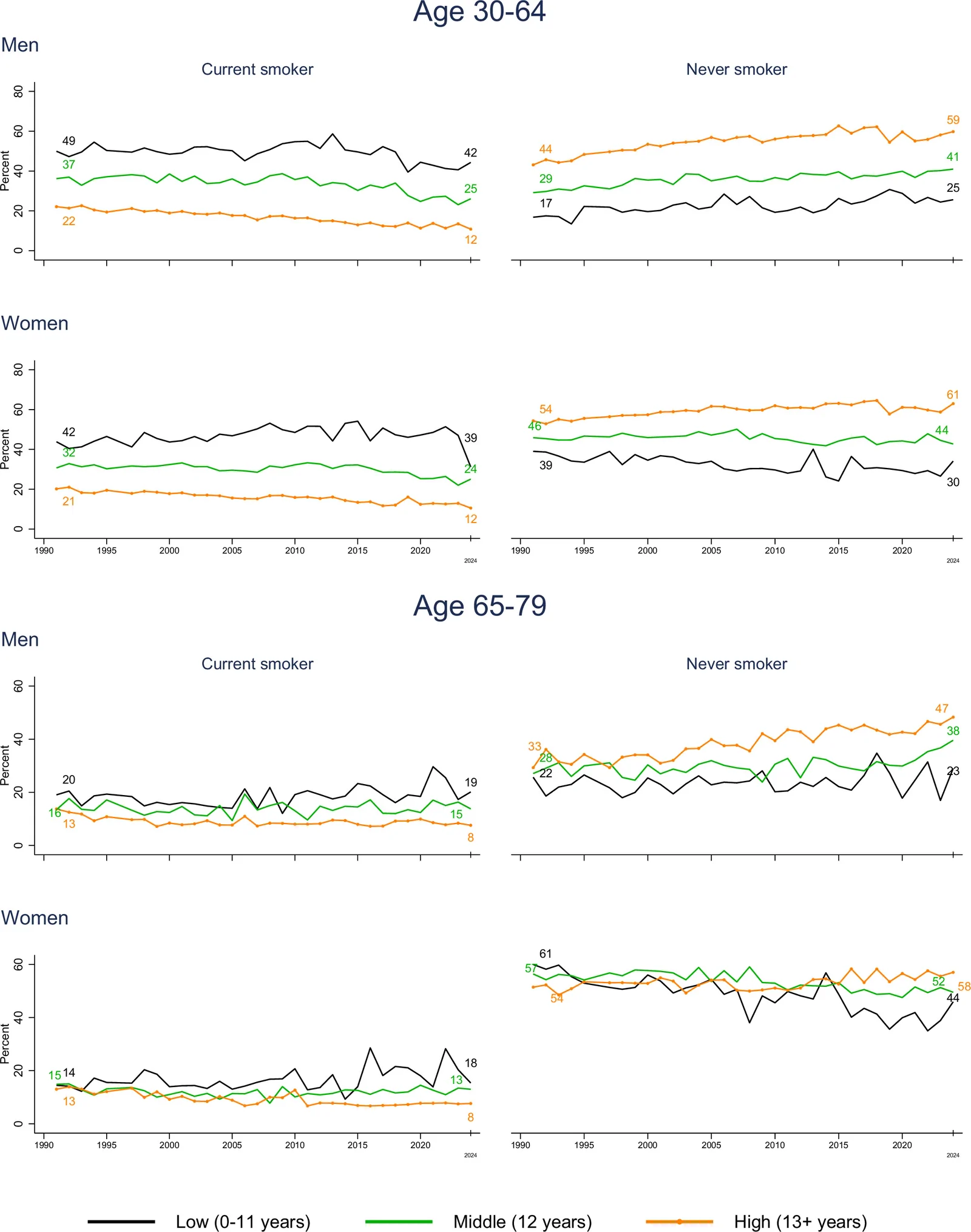
Percentage of current and never smokers in whites aged 30-64 and 65-79 by sex and education in the NHIS, 1990-2024. Notes: Numbers of current and never smokers are weighted by appropriate sampling weights specified by NHIS. White refers to Non-Hispanic white individuals. The two-year mean percentage of current and never smokers in the first and last two years are labeled on the plots.

From 1990 to 2024, the education gradient in the prevalence of current or never smoking widened substantially in the nationally representative National Health Interview Survey (NHIS)^20^. Among men, the gap between the high-education and low-education groups increased from 27 percentage points in 1990 to 30 percentage points in 2024 for current smoking and from 27 to 34 percentage points for never smoking (Extended Data Fig. 4). Among women, the corresponding gaps widened from 21 to 37 percentage points for current smoking and from 15 to 31 percentage points for never smoking. Notably, the prevalence of never smoking increased modestly among men with low education but declined over time among women with low education. The more pronounced divergence in never smoking, particularly among women, indicates that an increasing proportion of those with higher education have completely averted the long-latency mortality risks from smoking^21^. Extended Data Fig. 4 also shows the trends for current and never smoking among those aged 65–79 years.

### Addressing possible sources of bias in smoking-attributable mortality

Results from the indirect method used to determine smoking-attributable mortality rates depend on the smoker–never smoker RRs for various diseases, which we derived from the 2014 US Surgeon General’s Report (USSGR)^21^. If such risks were substantially larger in low-education groups, we would overestimate the contribution of smoking-attributable deaths to mortality differences by education. To assess this possible bias, we conducted pooled analyses of three large prospective studies in the USA, UK and Canada, which mostly comprise white populations of European descent with broadly similar chronic disease patterns and smoking trajectories^22^. Relying solely on the US NHIS yielded smaller and statistically less robust (albeit similar) estimates. Reassuringly, analyses of these three cohorts showed that smoker–never smoker hazard ratios (HRs) were generally similar across all three education levels for cardiovascular disease, lung cancer, other cancers, respiratory disease and all-cause mortality (Extended Data Table 1).

**Extended Data Fig. 5:**
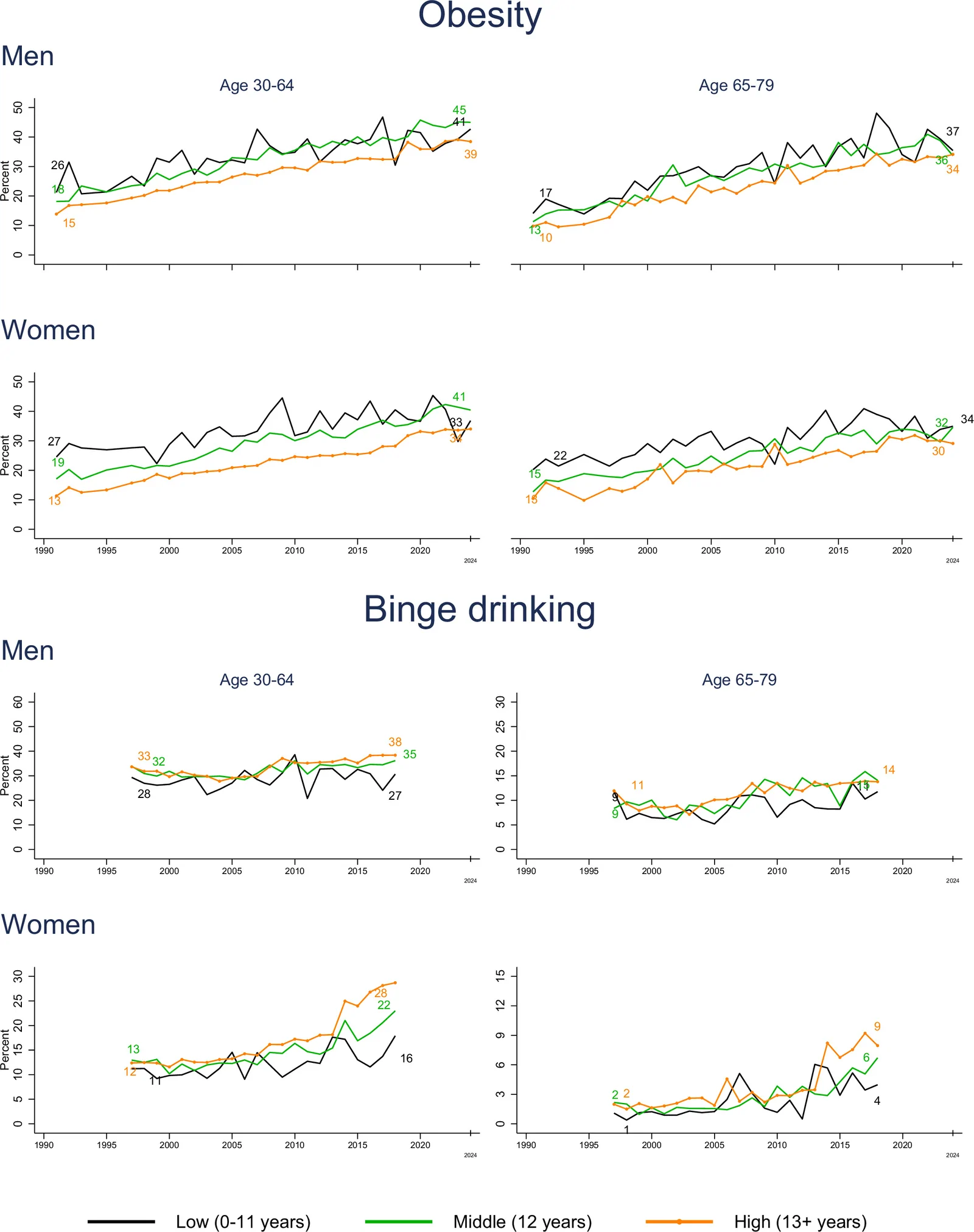
Percentage of people with obesity (Body Mass Index ≥ 30 kg/m^2^) and percentage of people who had five or more drinks in at least one occasion in the last 12 months in whites by age, sex and education in the NHIS, 1990-2024. Notes: Numbers of people with obesity are weighted by appropriate sampling weights specified by NHIS. White refers to Non-Hispanic white individuals. The two-year mean percentage of adults with obesity in the first and last two years are labeled on the plots. Data for binge drinking are available in 1997-2018.

In addition, the contribution of smoking to excess deaths could be biased if the smoker–never smoker HRs were differentially confounded by risk factors other than smoking in low- versus high-education groups. There was little evidence that potential confounding was greater in low-education groups for any of the major smoking-attributable diseases, despite the higher prevalence of smoking, obesity and excessive alcohol intake in these groups. We assessed potential confounding by examining changes in the log HR after adjusting for additional risk factors and found that the reductions in the log HR were generally small (10% or less). This suggests that if the measurement error in the imperfectly measured confounders were, in fact, much lower, it would only minimally reduce the observed strong association between smoking and disease. In addition, the trends by sex in obesity (defined as a body mass index (BMI) of at least 30 kg m^−^^2^) and binge drinking (defined as having at least five drinks on one occasion or more in the past 12 months) over time in the NHIS (Extended Data Fig. 5) showed little difference between education groups for each sex and age.

**Extended Data Fig. 6:**
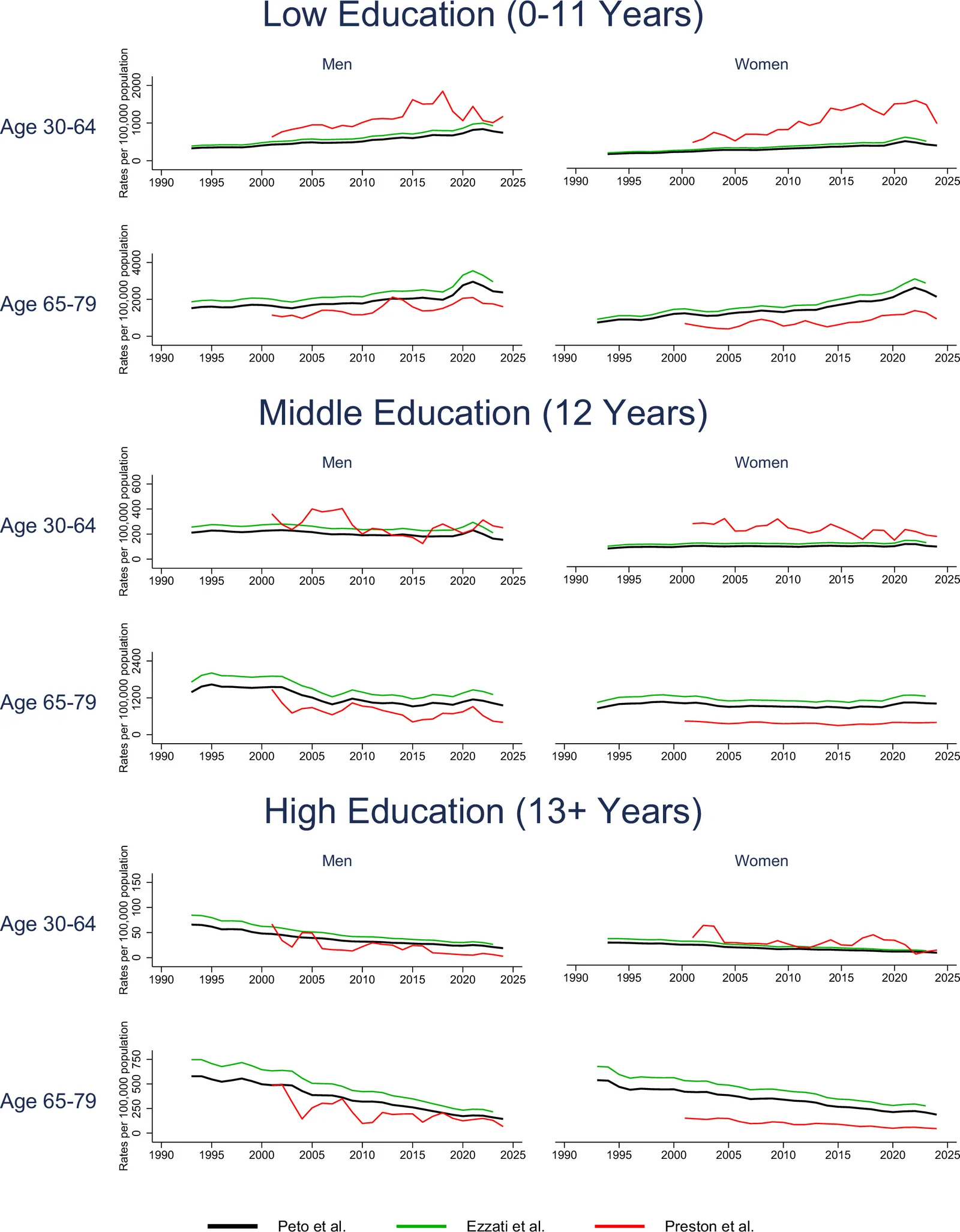
Sensitivity analysis on smoking deaths estimation of two-year moving average of smoking-attributable age-adjusted mortality rates in whites by age, sex and education groups, 1992-2024. Notes: Mortality rates adjusted to 2000 US standard population. White refers to Non-Hispanic white individuals. Ezzati-Lopez method reduces excess risk of smoking by 30%, rather than 50% in the Peto-Lopez method. Preston-Glei-Wilmoth (PGW) method estimates empirical coefficients (β) from regression analysis of 20 high-income countries that quantify the relationship between lung cancer mortality and mortality from other causes. These coefficients are then applied to observed excess lung cancer (above never-smoker rates) to estimate the fraction of deaths from other causes attributable to smoking, without requiring direct data on smoking prevalence. Most (if not all) of the 20 countries have near-universal healthcare access and better health outcomes than the US. Furthermore, PGW is only applicable for age 55+ and for years after 2000. § Y-axis scales for age group 65-79 are 2 times that of age group 30-64.

The indirect method subtracts the lung cancer rates of never smokers from the absolute lung cancer mortality rates, as shown in Figs. 3 and 4. Our graphed results based on the mortality data were consistent with analyses of lung cancer rates among never smokers in the three cohorts. These showed broadly similar lung cancer mortality among never smokers across education levels, which did not change appreciably over time in either sex (Extended Data Table 2), consistent with earlier analyses of the American Cancer Society cohorts^23^. Finally, we compared the method described by Peto et al.^18^ with two other indirect methods^24,25^ to quantify smoking-attributable mortality, with the Peto method yielding the most conservative and stable estimates (Extended Data Fig. 6).

### Contribution of various causes to excess deaths at ages 30–79 years

**Fig. 5:**
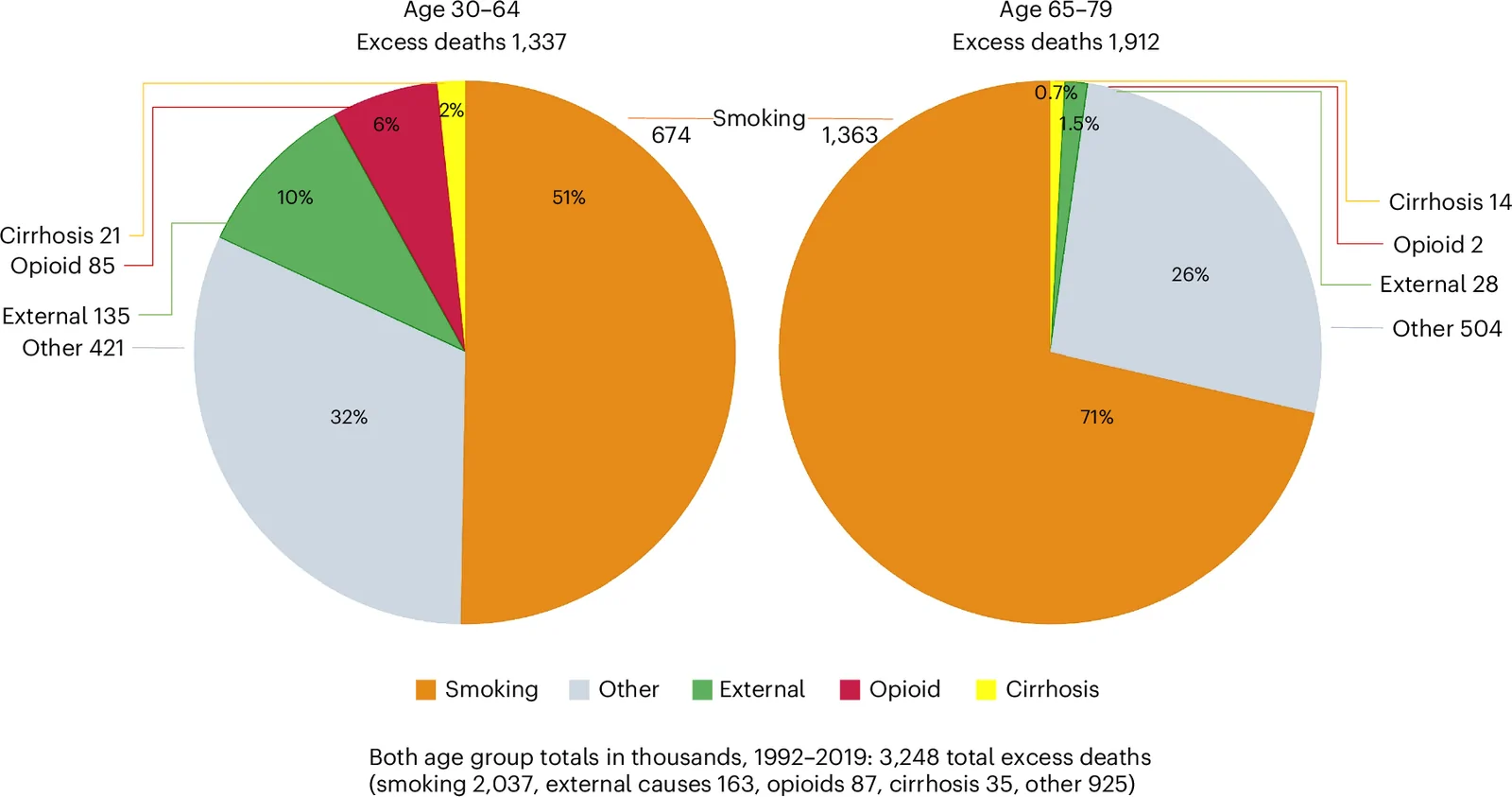
Excess deaths (in thousands) among low- and middle-education white adults aged 30–79, by contribution (%) of specific causes, 1992–2019. Results from 2020 to 2021 are not graphed to exclude COVID-19 effects. White refers to non-Hispanic white individuals. Deaths from ‘Other’ causes refer to those not attributable to smoking and not caused by opioids, cirrhosis, suicide, accidents or violence. Deaths from ‘External’ causes include those caused by suicides, accidents and violence, but not opioids. Excess deaths in thousands are displayed by cause and in totals by age group and time. Of the excess deaths from external causes, 12% were due to suicide at ages 30–64 and 5% at ages 65–79 during 1992–2019. Extended Data Table 3 provides more detailed tabulations of the contributions of smoking and other causes to age- and sex-specific mortality. Excess deaths are the difference between observed and counterfactual rates. Counterfactual death rates project the 1992–1993 mortality trend forward, assuming that the low- and middle-education groups experienced the same cause-specific rate of decline as the high-education group.

From 1992 to 2019, in both sexes, smoking-attributable diseases accounted for 63% of the excess 3.2 million deaths at ages 30–79 in the low- and middle-education groups (see Fig. 5, which provides the results for ages 30–64 and 65–79 years). Opioid poisoning, cirrhosis and external causes accounted for 3%, 1% and 5%, respectively. Other medical conditions contributed to the remaining excess deaths. Over this period, smoking accounted for 50% of excess deaths at ages 30–64 and 71% of excess deaths at ages 65–79 (Extended Data Table 3). The contributions of opioid poisoning, cirrhosis and external causes were much higher at ages 30–64 (6%, 2% and 10%, respectively) than at ages 65–79 (0.1%, 1% and 1%, respectively). From 2010 to 2019, the contributions of opioid poisoning and smoking to excess mortality at ages 30–64 were 8% and 45%, respectively.

## Discussion

The remarkable stagnation in survival among white adults in the USA is concentrated in low- and middle-education groups. At 2019 death rates, nearly four-fifths of low-education 30-year-old white men and two-thirds of white women are expected to die before age 80, a mortality rate comparable to the overall mortality observed during the Great Depression of the 1930s and in parts of sub-Saharan Africa today^26^. The lack of progress in reducing deaths at uniform rates across education groups equates to approximately 3.2 million excess deaths among low- and middle-education white adults over three decades.

The term ‘deaths of despair’ (ref. ^12^) has sparked debate on the drivers of stagnating mortality but has surprisingly ignored the role of smoking^4^. Our analyses confirm that smoking—an addictive behaviour influenced by economic and social despair—accounts for nearly two-thirds of the increase in educational inequalities in mortality in the USA, a proportion greater than that observed in cross-national analyses^27^.

The rising lung cancer rates in men and women with low education, despite declines in smoking prevalence (which measures a combination of starting and quitting), are somewhat perplexing. In the USA, smoking prevalence has declined in all socioeconomic groups, although less so among individuals with lower education and those over 50 years of age^16,21^. We noted a greater divergence between never and current smoking across education groups, especially among women. Lung cancer, other smoking-related cancers and COPD are strongly influenced by age of smoking onset, duration and type of smoking, and cumulative and lagged smoking exposure^21^. Therefore, the benefits of lifelong never smoking appear predominantly in highly educated individuals. The prevalence of never smoking declined in women but not in men. Thus, we would expect absolute lung cancer rates to decrease, at least among men with low education. Lung cancer is reliably reported on US death certificates and is not differentially reported by education group. Misreporting of education is also unlikely to be an explanation (see below)^28–30^. We note that never smoking and mortality have been linked in cross-sectional studies across US counties^10,31^.

Smoking is only a partial explanation for the divergence in mortality rates by education group, as age-adjusted mortality rates not attributable to smoking have risen for other cancers, COPD and, since about 2010, cardiovascular disease. Only with severe obesity (BMI ≥ 40 kg m^−^^2^) does the loss of life approximate the decade of life lost from smoking^32^. Obesity, while increasing in prevalence, does not by itself explain the divergence in mortality across education groups. However, obesity elevates blood pressure, raises adverse lipid profiles and causes diabetes, each of which is treatable^33^. Greater access to treatment by higher-education groups could have contributed to widening inequalities in mortality^34^.

It is highly improbable that the more speculative explanations—such as ultra-processed foods, pesticides, electromagnetic radiation, ambient air pollution, artificial food allergens, psychiatric drugs and microplastics, which have been suggested by the current US administration as predictors of childhood chronic diseases—have a role in mortality stagnation among low-education adults^35^. This is because the levels of exposure to these harmful agents are not increasing, or because exposures differ little across education groups^36–38^. Notably, Doll and Peto^39^ quantified the causes of cancer in the early 1980s in the USA and concluded that smoking alone accounted for a greater proportion of the cancer mortality risk than the sum of several environmental exposures. Indeed, attention to nonzero but small absolute risks from such exposures may confuse public understanding and divert policy attention from the largest avoidable risks, particularly those from smoking^40^.

This national analysis, based on vital statistics data, has the advantage of capturing overall population trends owing to the high coverage of death certification across education groups. However, there are some limitations. First, the proportion of low-education adults is declining, but current low-education adults differ from earlier cohorts in smoking patterns and social factors, including background access to healthcare^19^. The lung cancer-based index method implicitly captures the period and cohort effects of changes in group composition. However, we did not quantify subtle differences, such as the lower-education groups receiving fewer preventive services. For example, until about 2013, a considerable proportion of the population under age 65 lacked health insurance, especially adults with low education^41^. Indeed, we observed sex- and age-specific stagnation or increases in some conditions not attributable to smoking, and progress in survival even among highly educated US adults has been slower than overall progress in other countries (Extended Data Table 4). We did not quantify within-cohort compositional change: lower-educated individuals are more likely to die at younger ages, so low-educated survivors at older ages represent a positively selected group whose mortality burden we may have underestimated^42^. Overall, these unmeasured compositional processes suggest that our estimates of excess deaths among lower-educated white adults are conservative. Finally, comparing the mortality effects of long-term smoking exposure with the shorter-term mortality effects of opioids and alcohol-related injuries may involve subtleties of duration–dose response that our analyses did not address. A deeper examination of social and economic factors, including urban–rural differences, is warranted^10^. We plan to further examine trends by educational attainment among US Black adults and those of Hispanic ethnicity, in whom the smoker–nonsmoker HRs may well differ^8^.

Second, the indirect method used to estimate smoking-attributable mortality requires assumptions about the relationship between absolute lung cancer rates and smoker–nonsmoker RRs for other diseases. We found little evidence that we overestimated smoking-related mortality in lower-education groups or that the method has become biased over time. Third, there is probably misclassification of causes of death in the National Death Index, which could inflate smoking-attributable death rates for some conditions while lowering them for others, although it would not affect all-cause mortality^43^. These limitations highlight the need for further investigation in prospective studies and in nationwide mortality and demographic analyses of the contribution of smoking and other diseases to changes in survival, including in other countries.

In summary, smoking-attributable mortality is the major contributor to stagnating survival rates among the US white population, particularly in lower-education groups. Deaths from opioid-related and other external causes have increased over the past decade. Stabilization or modest declines in smoking-attributable lung cancer and COPD beginning around 2015 (refs. ^44,45^), along with recent declines in opioid mortality^46^, demonstrate that mortality reductions are possible. In particular, quitting smoking by age 40 avoids about 90% of the risk associated with continued smoking, and approximately half of the mortality benefits of long-term smoking cessation accrue rapidly within the first 3 years after quitting^22^. Absolute increases in smoking cessation among low-education groups would yield the largest gains in population health and offer the best intervention to reverse the remarkable stagnation of survival among white people in the USA.

## Methods

### US mortality and population data

We included the entire US white population from 1992 to 2024. We used US national mortality data, which have captured the education level of deceased individuals since 1989 (ref. ^47^). We obtained the underlying population data by age, sex and education level from the Current Population Survey (CPS)^48^. We focused on trends from 1992 to 2019 because education reporting had noticeable gaps from 1989 to 1991. We chose 2019 to avoid the 2020–2021 mortality spikes caused by COVID-19, which sharply increased overall mortality, particularly in the lower-education groups^49^.

### Age, ethnicity and education definitions

We chose 30 years as the starting age for our analyses to allow sufficient time for most individuals to complete their college education. We used the Medicare eligibility age of 65 years as the lower cut-off for the older adult age group because Medicare has covered nearly all US older adults since the 1960s, and insurance coverage may alter mortality risks^50^. We used 79 years as the upper age in our analyses, as this exceeds the most recent median life expectancy in the USA. Our focus is on premature deaths, and the median life expectancy is a good, albeit arbitrary, standard for defining them.

US death certificates indicate whether the decedent was white, Black/African American or from other racial categories. A separate question determines Hispanic ethnicity. We defined white people as non-Hispanic white individuals. Death certificates recorded the decedents’ years of education completed at the time of death. We examined three education levels: ≤11 years (low-education group), 12 years (middle-education group) and ≥13 years (high-education group). We conducted sensitivity analyses using 16 or more years of education as a comparison (Extended Data Fig. 1). We excluded 1.5 million white decedents aged 15–79 with unknown education levels between 1992 and 2024.

A potential source of bias arises from the use of death certificate data, which rely on proxy reporting of educational attainment by family members or funeral directors, whereas the population denominator is based on self-reported education from the CPS. The primary documented discrepancy between these sources is the misclassification of individuals with less than a high school education as high school graduates on death certificates^29^. Such misclassification has declined over time^30^ and, therefore, is unlikely to explain the observed increases in mortality among the least educated. If anything, this bias would lead to an underestimation of the increase in mortality among low-education groups. Moreover, this misclassification is unlikely to materially affect our estimates of excess deaths from smoking, opioids and other causes, as this analysis compares mortality in the two lower-education groups with that in the highest-education group^28^. Finally, we noted little or no difference when excluding states with low completeness of education data in the early years after education was added to death certificates (Supplementary Fig. 1).

### Causes of death

We used the Mortality Multiple Cause Files to generate rates and risks. During our study period, nearly 100% of deaths were medically certified, with causes assigned according to the ICD-9 (up to 1998) and ICD-10 (1999 onward) systems^51^.

### Smoking, obesity and binge drinking prevalence

We used the NHIS^52^ to estimate the percentage of adults who were current smokers (smoking every day or some days), never smokers (having never smoked 100 cigarettes in their lifetime) or had obesity (BMI ≥ 30 kg m^−^^2^) from 1990 to 2024. We defined binge drinking as having five or more drinks on at least one occasion in the past 12 months and estimated the percentage of adults who engaged in binge drinking from 1997 to 2018. We applied the appropriate sampling weights specified in the NHIS.

### Statistical methods

#### Rates and risks of death

The risk of death at ages 30–79 years applies a uniform averaging of the age-specific rates (*R*_*p*_; per 100,000 population) across the ten 5-year age groups (30–34, 35–39, …, 75–79). The risk of death between ages 30 and 79 years is thus 1 − exp(−50 *R*_*p*_/100,000). Risks are not subject to variation in the population composition in any given year. Rates are standardized to the 2000 US standard population.

#### Excess all-cause mortality

For each age group and sex, we estimated the average annual rate of reduction (AARR) separately for each education group. Using 1992 and 1993 separately as base years, we regressed log(mortality) on elapsed time since the base year (*t* = year − base year) over the 1992–2019 time period, yielding two estimates of ${\hat{\beta}}$ per group. Each $\hat{\beta }$ was converted to an AARR using the formula $\mathrm{AARR}=1-\exp ({\hat{\beta}})$ (ref. ^53^). To estimate excess deaths (that is, deaths that could have been prevented had the low- and middle-education groups experienced the same mortality decline as the high-education group (*m̂*_*t*_)), we projected counterfactual death rates using the high-education group’s $\overline{\mathrm{AARR}}$ applied to each group’s own baseline rates:

$${\hat{m}}_{t}={\bar{m}}_{\mathrm{base}}\times {(1-\overline{\mathrm{AARR}})}^{\tau },$$

where ${\bar{m}}_{\mathrm{base}}$ is the mean of the 1992 and 1993 age-standardized death rates for the low- or middle-education group; $\overline{\mathrm{AARR}}$ is the mean of the AARRs for the highest-education group, estimated using 1992 and 1993 as separate base years; and *τ* is the mean of (*t* − 1992) and (*t* − 1993)—that is, the average elapsed time from each base year.

#### Indirect method for smoking-attributable deaths

The Peto method is a widely used indirect approach for estimating smoking-attributable deaths across multiple causes (for example, cardiovascular, respiratory and neoplastic diseases)^25^. The Peto method approximates cumulative population exposure to smoking in settings where reliable survey data on lifetime smoking patterns are unavailable. It leverages the fact that lung cancer deaths reflect long, lagged smoking histories—typically cumulative exposure 20–30 years earlier, although the lag is shorter at younger ages. Because of this lag, observed lung cancer death rates incorporate cohort effects (such as when birth cohorts began smoking and at what intensity) and period effects (including policy shifts, tobacco availability and cessation campaigns). The Peto method does not explicitly model age, period or cohort effects but instead captures them indirectly: age effects by computing the smoking impact ratio (SIR) within age groups (older age groups reflect longer cumulative exposure and higher lung cancer mortality risk); cohort effects through age-specific lung cancer death rates (cohorts with more intensive early-life smoking show excess lung cancer deaths in the numerator); and period effects through observed lung cancer death rates from a given calendar period (changes in lung cancer risk from factors such as reduced tar content shift the measured rates). Briefly, we followed a multistep approach.

##### Step 1. Calculate the observed lung cancer death rates

First, we calculated absolute age- and sex-specific lung cancer mortality rates at ages 30–64 and 65–79 years (smoking causes few deaths before age 30)^40^ for each education group using the Mortality Multiple Cause Files^47^ death data and CPS^48^ population estimates.

##### Step 2. Calculate the SIR

Second, we calculated the SIR using our observed death rates (as noted in step 1) and the reference rates from a large 1980s prospective study of 1.2 million Americans (ACS-CPS II)^23^. The SIR numerator is the difference between the annual age-specific lung cancer death rates and the reference lung cancer death rates for nonsmokers. The denominator is the difference between the smokers’ and nonsmokers’ reference lung cancer death rates. This difference eliminates shared reasons for developing lung cancer among smokers and nonsmokers. Thus, the SIR represents lung cancer deaths caused solely by smoking:

$$\mathrm{SIR}=\frac{C-N}{S-N},$$

where *C* is the age-specific lung cancer death rate of the observed population, *S* is the lung cancer death rate of reference smokers from the ACS-CPS II (see appendix in ref. ^18^), and *N* is the lung cancer death rate of reference nonsmokers from the ACS-CPS II (see appendix in ref. ^18^).

While, mathematically, the SIR could be <0 or >1, we bounded the SIR between 0 and 1, with 0 representing smoking having no contribution to mortality and 1 representing smoking completely contributing to death in this group.

##### Step 3. Estimate smoking-attributable mortality for other causes

We used the SIR to estimate the contribution of smoking to other causes of death, including other cancers, respiratory diseases, cardiovascular disease and certain other conditions (Extended Data Table 1 details which causes were attributed to smoking; deaths from cirrhosis and nonmedical causes were not attributed to smoking). We applied age- and sex-specific smoker–never smoker RRs for various conditions from the 2014 USSGR^21^, which provides the most current RRs for all major smoking-attributable conditions. The excess risk of death from smoking incorporates both the SIR and the RRs:

$$\mathrm{Excess}\,\mathrm{risk}\,\mathrm{of}\,\mathrm{death}\,\mathrm{from}\,\mathrm{smoking}=\frac{\mathrm{SIR}\times \left(\mathrm{RR}-1\right)}{2}.$$

To account for potential confounding—smokers may be more likely to drink alcohol, have obesity or have other risk factors—we applied an arbitrary 50% reduction to the excess risk for conditions other than lung cancer^23^. For example, the USSGR^21^ reports a male smoker–never smoker RR of 3.0 for coronary heart disease at ages 55–64 years. The excess risk of 2.0 becomes 1.0 after the 50% adjustment. Earlier sensitivity analyses showed that varying this correction factor from 0% to 60% had little effect on the results^25^.

##### Step 4. Calculate the proportion of smoking-attributable deaths

The proportion of smoking-attributable deaths (PSAD) follows directly from the excess risks:

$$\mathrm{PSAD}=\frac{\mathrm{Excess}\,\mathrm{risk}}{1+\mathrm{Excess}\,\mathrm{risk}}.$$

##### Step 5. Derive smoking-attributable death rates

Finally, we multiplied the PSAD by age- and sex-specific mortality rates to derive estimated smoking-attributable deaths for the causes of interest:

$$\begin{array}{ll}\mathrm{Age} {\hbox{-}}{\mathrm{specific}}\,{\mathrm{smoking}}{\hbox{-}}{\mathrm{attributable}}\,{\mathrm{death}}\,{\mathrm{rate}} \\ \quad \quad ={\mathrm{age}}{\hbox{-}}{\mathrm{specific}}\,{\mathrm{death}}\,{\mathrm{rate}}\times {\mathrm{PSAD}}.\end{array}$$

We standardized overall and cause-specific mortality rates for ages 30–79 (and subgroups such as ages 30–64 and 65–79) to the 2000 US standard population.

#### Assessing potential confounding by education

Smoking, obesity and excessive drinking are more prevalent in lower-education groups than in higher-education groups in the USA. To assess whether confounding by smoking differed by education level and disease type, we examined smoker–never smoker HRs across education groups for major diseases using Cox proportional hazards models. We analysed data from three prospective studies: the US NHIS linked to the National Death Index (1997–2004), the UK Biobank study (2006–2010) and the Canadian Community Health Survey (2000–2014)^52,54,55^. These three studies—broadly similar in demographic structure and smoking patterns—produce more stable estimates than the NHIS alone (Supplementary Table 1).

We computed stratum-specific smoker–never smoker HRs and compared the log HRs adjusted for age alone with the log HRs adjusted for age, obesity and excessive drinking (Extended Data Table 2). Substantial residual confounding would markedly reduce the log HR^22^. Additionally, because the indirect method subtracts the lung cancer rates of never smokers, we assessed whether these background rates varied by education or changed over time.

#### Opioid poisoning, cirrhosis and external mortality

We defined opioid deaths using the US Centers for Disease Control and Prevention classification for illicit and prescription-based opioid poisoning, which specifies deaths involving opioid use as a contributory cause using the ICD-10 T-codes or as an external cause of injury using ICD-9 E-codes (Supplementary Table 2).

Extended Data Table 4 provides the contributions of smoking, opioids and other causes, separately for the low- and middle-education groups, by sex and age group. Our main analysis does not include unspecified drugs in opioid deaths. To investigate the extent to which unspecified opioid deaths could affect our results, we included all unspecified deaths (that is, those with ICD-10 code T50.9 but no other specified drugs) as opioid deaths for ages 30–64 and 65–79 years (Supplementary Fig. 2). Our main results remained unchanged with this procedure. Although excluding unspecified drugs may underestimate opioid deaths, we have no evidence that all or the majority of these unspecified drugs are indeed opioids. Similarly, our definition of opioid mortality may underestimate suicides as the underlying cause of death.

We quantified, for each education stratum, the contributions of opioid poisoning, cirrhosis and external causes to mortality. We subtracted deaths from smoking-attributable causes, opioid poisoning, cirrhosis and external causes from the total mortality in each age-, sex- and education-specific stratum to determine deaths from all other causes. All analyses were conducted in Stata 17 (ref. ^56^) and SAS 9.4 (ref. ^57^).

### Ethics and inclusion

The study used publicly available, de-identified data on mortality and risk factors, and no ethical permission was needed.

### Reporting summary

Further information on research design is available in the Nature Portfolio Reporting Summary linked to this article.

## Supplementary information

Supplementary Information Supplementary Tables 1 and 2 and Figs. 1 and 2.

Reporting Summary

Peer Review File

## Figures and Tables

**Extended Data Table 1:**
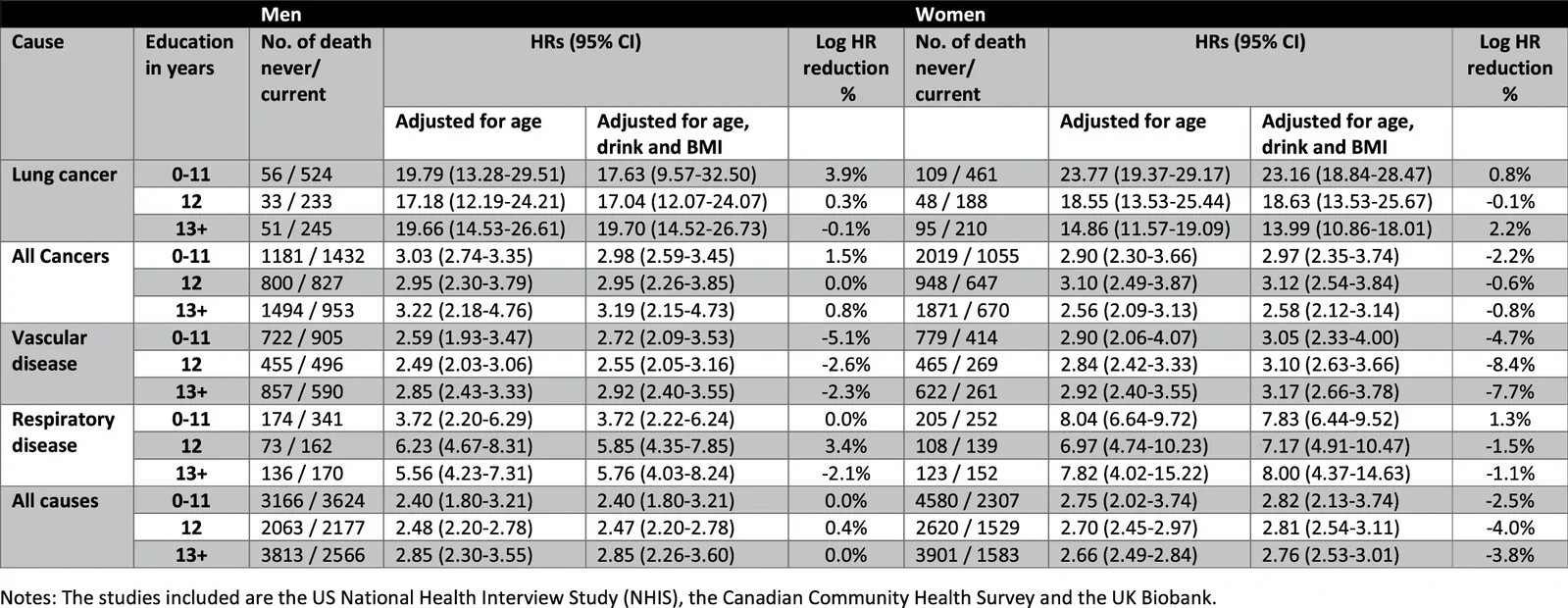
Pooled Hazard Ratios (HR) for smoking and cause-specific mortality in three prospective studies by sex, causes and education level, and reduction in the log HR with adjustment for known confounders

**Extended Data Table 2:**
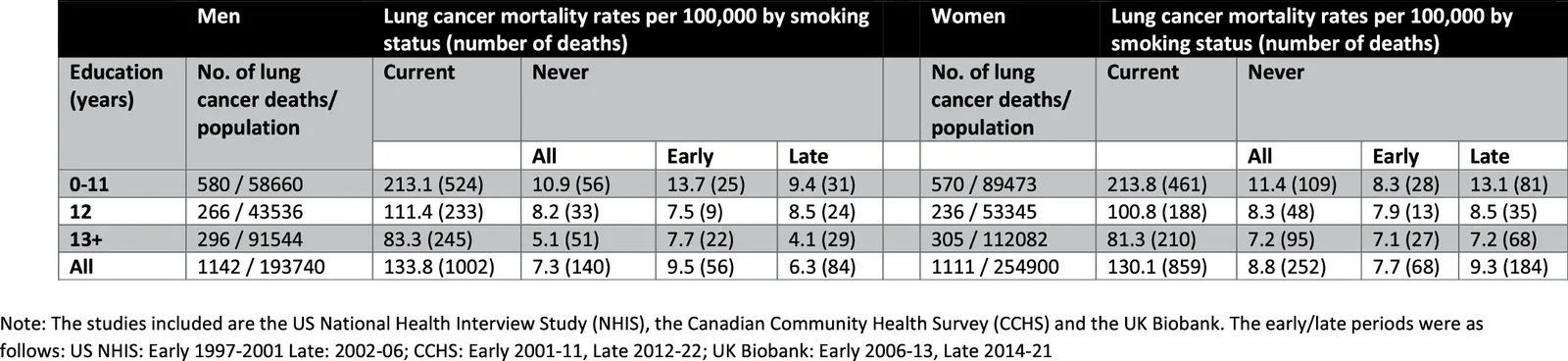
Pooled lung cancer mortality rates (per 100,000) in three prospective studies in current and never smokers by education level and during the earlier and later periods of observation

**Extended Data Table 3:**
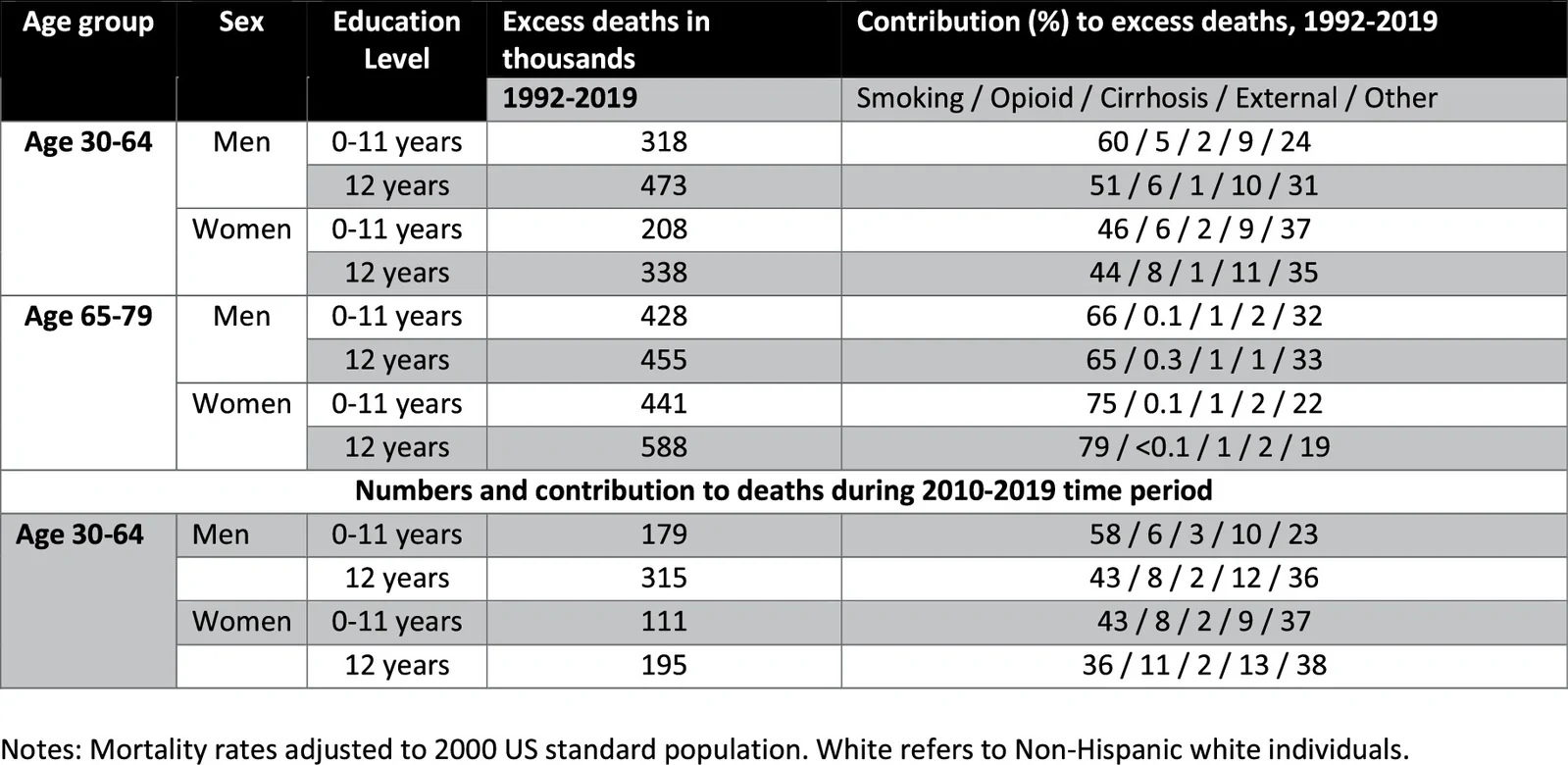
Age-adjusted all-cause mortality rates (per 100,000) and cause-specific contribution to mortality in whites aged 30-64 and 65-79 by sex and education from 1992-2019

**Extended Data Table 4:**
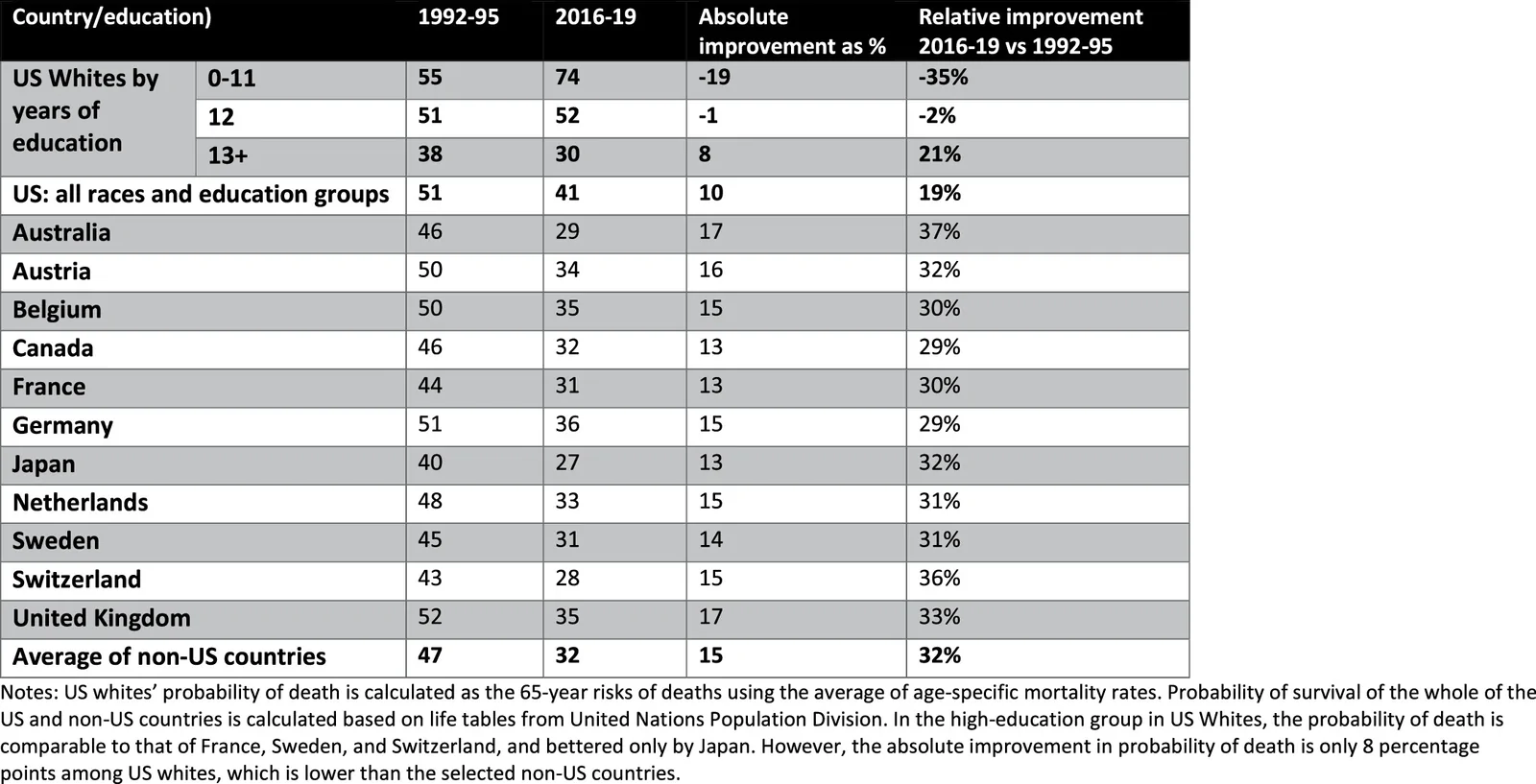
Comparisons of probability of death at ages 15-79 among US whites by education and selected other countries, 1992-96 vs. 2016-19 both sexes combined

## Data Availability

The datasets used are publicly available from the National Vital Statistics System. The UK Biobank, Canadian Community Health Survey and US National Health Interview Survey (NHIS) data are also publicly available. US death records data are available through the Vital Statistics Online Data Portal at https://www.cdc.gov/nchs/data_access/vitalstatsonline.htm#Mortality_Multiple. The population data are available in the Annual Social and Economic Supplements of the Current Population Survey at https://www.census.gov/data/datasets/time-series/demo/cps/cps-asec.html. The reference death rates by cause and smoking history are available in the appendix of Peto et al.^18^ at https://doi.org/10.1016/0140-6736(92)91600-D. The risk ratios by cause and smoking history are available in Table 12.3 of the 2014 US Surgeon General’s Report at https://archive.cdc.gov/www_cdc_gov/tobacco/sgr/50th-anniversary/index.htm. For the results from the Cox proportional hazards models, the NHIS-linked mortality data are available at https://www.cdc.gov/nchs/linked-data/mortality-files/?CDC_AAref_Val. Data from the Canadian Community Health Survey are available at https://www23.statcan.gc.ca/imdb/p2SV.pl?Function=getSurvey&Id=1531795. Data from the UK Biobank are available at https://www.ukbiobank.ac.uk/. A table of age and sex-specific death counts by education can be generated using the Stata code available on Code Ocean (https://doi.org/10.24433/CO.5179147.v1).

## Code availability

The Stata code for this study is available on Code Ocean (https://doi.org/10.24433/CO.5179147.v1) and from P.J. (Prabhat.jha@ndph.ox.ac.uk) at the University of Oxford upon reasonable request within 6 months of receipt.
