# Supplementary material for: Smoking, education level and excess premature deaths in the USA
Source: Nat Health. 2026 Jul 10;1(11):1235–46. doi: 10.1038/s44360-026-00153-7 (PMC13727667; doi:10.1038/s44360-026-00153-7)
Supplement: Supplementary file 1 [file 44360_2026_153_MOESM1_ESM.pdf]

nature health

Supplementary information https://doi.org/10.1038/s44360-026-00153-7

Smoking, education level and excess premature deaths in the USA

In the format provided by the authors and unedited

Supplementary Material

Supplementary Table 1. Sample characteristics of US NHIS, Canada CCHS and UK Biobank and variables used

	US NHIS	Canada CCHS	UK Biobank
**Age**	30-79	30-79	40-73
**Race**	white (100% in this sub-study)	white (83.7%) Asian (8.4%) Black (1.9%) Other (6.0%)	white (93.3%) Asian (2.8%) Black (2.0%) Others (1.9%)
**Survey years**	1997-2018	2000-2014	2006-2010
**Mortality follow-up years**	1997-2019 (lung cancer 1997-2006)	2000-2022	2006-2021
**No. Subject (men/women)**	83053/100091	27894/34991	125157/170028
**No. Death (men/women)**	5682/6127	2451/2472	9276/7921

Variable definitions for hazard analysis

Variables	Form	Categories	Definition
**age**	continuous		
**drink**	categorical (4 group)	Never / Former / infrequent and light / moderate and heavy	infrequent and light (≤3 times/week);<br>moderate and heavy (>3 times/week)
**Body mass index (BMI)**	categorical (3 group)	<25 / 25-<30 / 30+	underweight or healthy weight (BMI < 25);<br>overweight but not obese (25 ≤ BMI < 30);<br>obese (BMI ≥ 30)

Notes: For ICD-9 codes specifying death caused by opioids, the record may have an N code in the Diagnosis column or an E code in the External Cause of Injury column. For ICD-10 codes specifying death caused by opioids, the record need to have an Underlying Cause code and a Contributing Cause code from the ones listed in each column. Definition of opioid-related deaths based on "Using International Classification of Diseases (ICD) Codes to Assess Opioid-Related Overdose Deaths. Substance Abuse and Mental Health Services Administration; 2018." For hazard analysis, former smokers who quit within 5 years before death are defined as current smokers. Hazard analysis excludes former smokers, those with a history of vascular disease, cancer or respiratory disease, or those with missing causes of death, years of education, smoking status, drinking status and BMI. We excluded individuals with missing smoking status, history of chronic disease (diabetes, hypertension, vascular disease, and respiratory disease), education level, BMI and alcohol status. In these survival analyses, we censored individuals according to the date of death or the end of follow-up, whichever came first. Deceased with missing education are excluded from analysis.

Supplementary Table 2. Definitions of Causes of death defined by ICD-9 and ICD-10 codes

Causes (* are those attributable to smoking)	ICD-9 (1992-1998)		ICD-10 (1999-2024)	
	Diagnosis	External Cause of Injury	Underlying Cause	Contributing Cause
**Lung Cancer***	162		C33-C34	
**Cancers***			C00-C97	
**Respiratory disease***	034.0, 460-519		J00-J99	
**Vascular disease (cardiovascular)***	390-459		I00-I99	
**Opioids**	965.00-965.02, 965.09	E850.0-E850.2	X40-X44, X60-X64, X85, Y10-Y14	T40.0-T40.4, T40.6 (subset of those where underlying cause was opioid deaths)
**Chronic liver disease and cirrhosis**	571		K70, K73-K74	
**Suicide**		E950-959	*U03, X60-X84, Y87.0	
**Assault**		E960-E978	*U01-*U02, X85-Y09, Y87.1	
**Accidental falls**		E880-E888	W00-W19	
**Accidental poisoning (excluding opioids)**		E850-E869, minus opioids	X40-49, minus opioids	
**Motor vehicle accidents**		E810-E825	V02-V04, V09.0, V09.2, V12-V14, V19.0-V19.2, V19.4-V19.6, V20-V79, V80.3-V80.5,V81.0-V81.1, V82.0-V82.1, V83-V86, V87.0-V87.8, V88.0-V88.8, V89.0, V89.2	
**All external causes**		E800-E999	V01-Y89	

Supplementary Figure 1. Sensitivity analysis on education completeness (includes only states with 80% completeness or more) of two-year moving average of age-standardized all-cause mortality rates in whites by age groups and years of education, 1992-2024

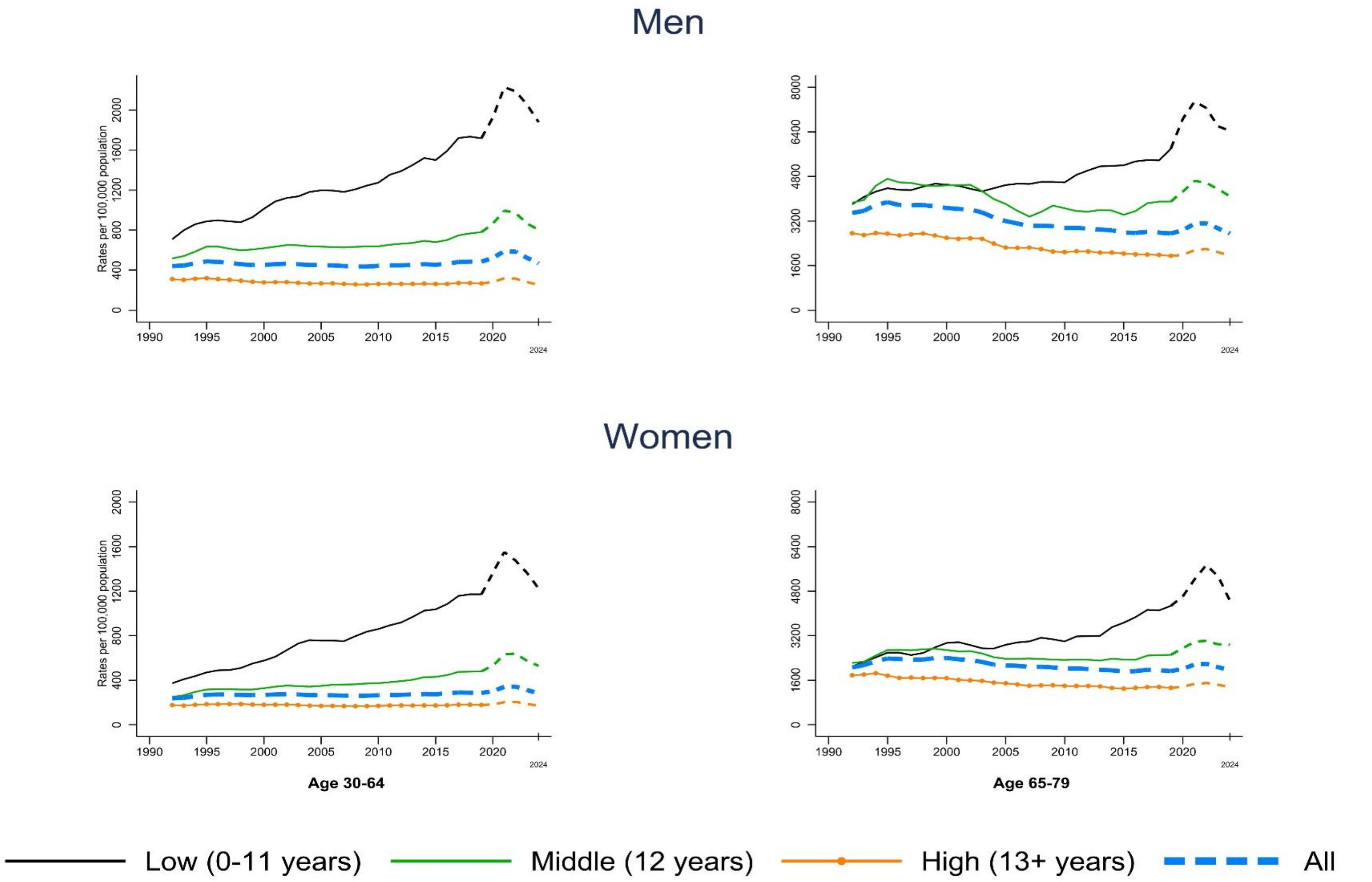

Notes: Mortality rates adjusted to 2000 US standard population. White refers to Non-Hispanic white individuals. Mortality affected by COVID-19 pandemic in dotted line. Y-axis scales for age group 65-79 are 4 times that of age group 30-64.

Supplementary Figure 2. Sensitivity analysis on opioid definition of two-year moving average of age-standardized cause-specific mortality rates in white men and women aged 30-64 & 65-79 by years of education, 1992-2019

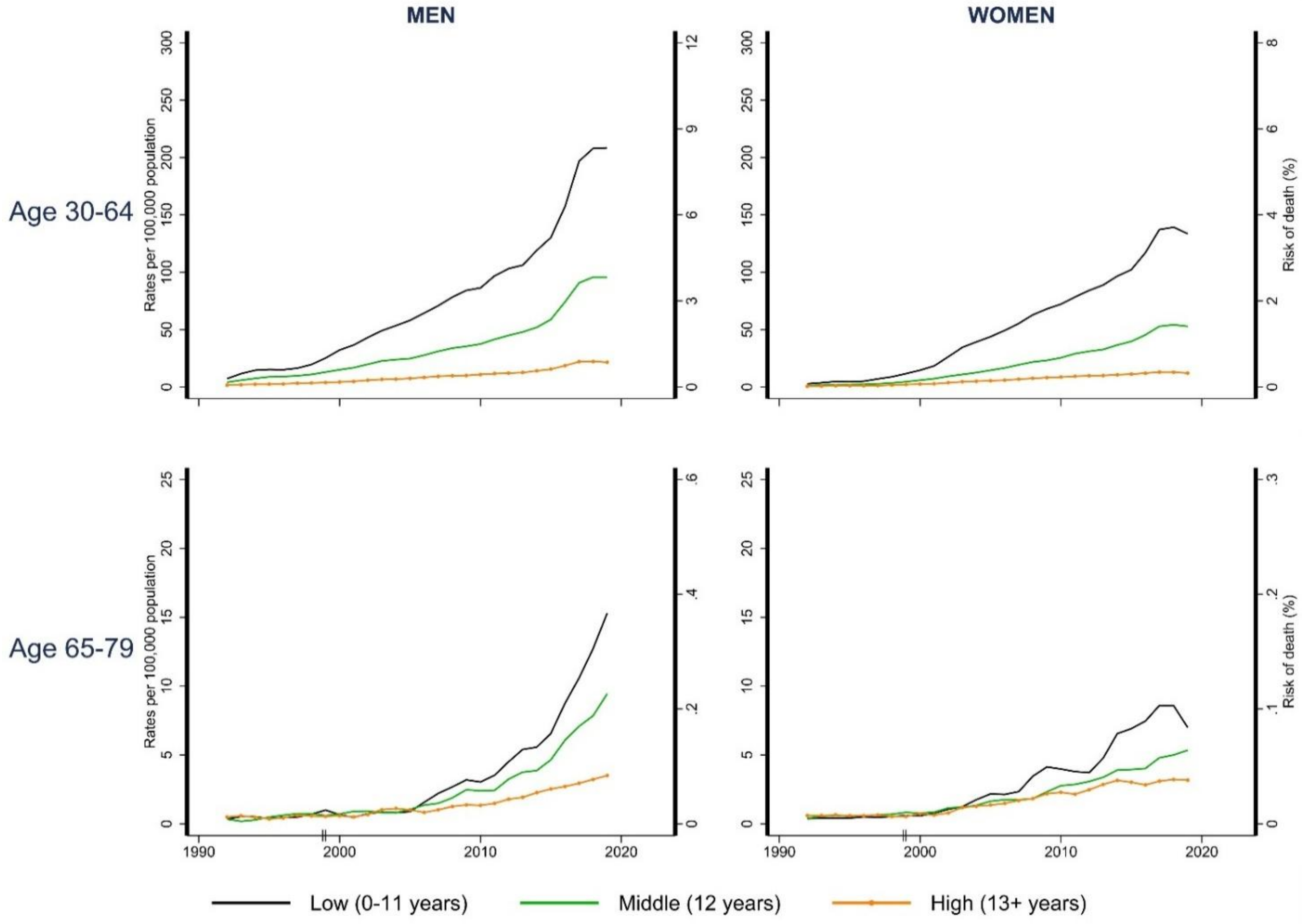

Notes: Mortality rates adjusted to 2000 US standard population. White refers to Non-Hispanic white individuals. Double bars on the x-axis reflect the change from ICD-9 to ICD-10 in 1999. Sensitivity analsis includes data with unspecified drugs which may be opioids. Either the ICD-9 diagnosis or external cause of injury codes could specify opioid deaths. In ICD-9, because both the nature of injury and external causes of injury codes encompasses the same range (nature of injury 800-999, external cause of injury E000-E999), the last digit (i.e. the 7th) of the entity codes is a 0 or 1 flag variable with 1 representing the nature of injury code. In the ICD-10, both the underlying cause and contributing cause codes were necessary to define opioid deaths.
