# Supplementary material for: Smoking, education level and excess premature deaths in the USA
Source: Nat Health. 2026 Jul 10;1(11):1235–46. doi: 10.1038/s44360-026-00153-7 (PMC13727667; doi:10.1038/s44360-026-00153-7)
Supplement: Supplementary file 2 [file 44360_2026_153_MOESM2_ESM.pdf]

nature portfolio

Corresponding author(s): Prabhat Jha

Last updated by author(s): May 11, 2026

Reporting Summary

Nature Portfolio wishes to improve the reproducibility of the work that we publish. This form provides structure for consistency and transparency in reporting. For further information on Nature Portfolio policies, see our Editorial Policies and the Editorial Policy Checklist.

Statistics

For all statistical analyses, confirm that the following items are present in the figure legend, table legend, main text, or Methods section.

n/a	Confirmed	
☒	☐	The exact sample size (*n*) for each experimental group/condition, given as a discrete number and unit of measurement
☒	☐	A statement on whether measurements were taken from distinct samples or whether the same sample was measured repeatedly
☒	☐	The statistical test(s) used AND whether they are one- or two-sided<br>*Only common tests should be described solely by name; describe more complex techniques in the Methods section.*
☐	☒	A description of all covariates tested
☐	☒	A description of any assumptions or corrections, such as tests of normality and adjustment for multiple comparisons
☐	☒	A full description of the statistical parameters including central tendency (e.g. means) or other basic estimates (e.g. regression coefficient) AND variation (e.g. standard deviation) or associated estimates of uncertainty (e.g. confidence intervals)
☒	☐	For null hypothesis testing, the test statistic (e.g. *F*, *t*, *r*) with confidence intervals, effect sizes, degrees of freedom and *P* value noted<br>*Give P values as exact values whenever suitable.*
☒	☐	For Bayesian analysis, information on the choice of priors and Markov chain Monte Carlo settings
☒	☐	For hierarchical and complex designs, identification of the appropriate level for tests and full reporting of outcomes
☒	☐	Estimates of effect sizes (e.g. Cohen's *d*, Pearson's *r*), indicating how they were calculated

Our web collection on statistics for biologists contains articles on many of the points above.

Software and code

Policy information about availability of computer code

Data collection	We did not use specific codes to collect data.
Data analysis	All analyses were conducted in Stata 17 and SAS 9.4. Stata codes are available on Code Ocean (DOI 10.24433/CO.5179147.v2).

For manuscripts utilizing custom algorithms or software that are central to the research but not yet described in published literature, software must be made available to editors and reviewers. We strongly encourage code deposition in a community repository (e.g. GitHub). See the Nature Portfolio guidelines for submitting code & software for further information.

Data

Policy information about availability of data

All manuscripts must include a data availability statement. This statement should provide the following information, where applicable:

- Accession codes, unique identifiers, or web links for publicly available datasets
- A description of any restrictions on data availability
- For clinical datasets or third party data, please ensure that the statement adheres to our policy

The datasets used are publicly available at the National Vital Statistics System. The UK Biobank, CCHS and US NHIS are also publicly available.

The US death records data are available in the Vital Statistics Online Data Portal at https://www.cdc.gov/nchs/data_access/vitalstatsonline.htm#Mortality_Multiple. The population data are available in the Annual Social and Economic Supplements of the Current Population Survey at https://www.census.gov/data/datasets/time-

series/demo/cps/cps-asec.html. The reference death rates by causes and smoking history are available in the appendix of Peto et al. (1992) at https://doi.org/10.1016/0140-6736(92)91600-D. The risk ratios by causes and smoking history are available in Table 12.3 of the US Surgeon General's Report (2014) at https://www.cdc.gov/tobacco/sgr/50th-anniversary/index.htm. For the results from the Cox-proportional hazard models, the National Health Interview Survey linked mortality data are available at https://www.cdc.gov/nchs/data-linkage/mortality-public.htm. Data from the Canadian Community Health Survey are available at https://www23.statcan.gc.ca/imdb/p2SV.pl?Function=getSurvey&Id=1531795. Data from the United Kingdom Biobank are available at https://www.ukbiobank.ac.uk/.

A table of age and sex-specific death counts by education can be generated using the Stata code available in Code Ocean.

Research involving human participants, their data, or biological material

Policy information about studies with human participants or human data. See also policy information about sex, gender (identity/presentation), and sexual orientation and race, ethnicity and racism.

Reporting on sex and gender	We use the term "sex" only in our manuscript. Findings apply to both "male" and "female" as reported on national surveys and US death certificates. We analyze data and report findings from "male" and "female" separately because "male" and "female" have different risks of death with "females" more likely to survive to older ages than "males". Sex-specific data came from the same data sources described in the Data Availability Statement.
Reporting on race, ethnicity, or other socially relevant groupings	Rather than using race/ethnicity as a variable, we stratified our sample to include White (Non-Hispanic White) Americans only. As this study's central aim is to examine mortality risks in the last 30 years in the US, Non-Hispanic White (hereafter 'White') populations, who comprise approximately 80% of the US population in 1990 and 60% in 2020 exhibit the greatest stagnation in survival. By contrast, survival of US adult blacks has improved and survival patterns of US Hispanics is strongly influenced by in migration of healthier cohorts. We plan future examination of trends in lower education US Black and Hispanic populations, in whom the smoker: non-smoker hazards may well differ.
Population characteristics	N/A
Recruitment	N/A
Ethics oversight	The study used publicly available, de-identified data on mortality and risk factors, and no ethical permission was needed.

Note that full information on the approval of the study protocol must also be provided in the manuscript.

Field-specific reporting

Please select the one below that is the best fit for your research. If you are not sure, read the appropriate sections before making your selection.

☐ Life sciences ☒ Behavioural & social sciences ☐ Ecological, evolutionary & environmental sciences

For a reference copy of the document with all sections, see nature.com/documents/nr-reporting-summary-flat.pdf

Behavioural & social sciences study design

All studies must disclose on these points even when the disclosure is negative.

Study description	This is a retrospective population-based quantitative, descriptive epidemiological study of age-standardized rates and uniformly standardized risks among US non-Hispanic White adults by sex and education level over 30 years (1992-2024).
Research sample	All deaths among non-Hispanic White adults aged [30-79] years recorded in the US National Vital Statistics System (NVSS) multiple-cause-of-death files, 1992–2024. Population denominators from US Census. From 1992-2024, 33 million (M) deaths in US whites with known education status occurred at ages 30–79 years, of which 19M occurred in males and 14M in females; 13M occurred at ages 30–64; and 20M occurred at 65–79 years. Sample is representative of the US population.
Sampling strategy	NA — full population census of US death records; no sampling was performed.
Data collection	We conduct analysis of secondary, de-identified data, and thus do not collect data ourselves. Death records are compiled from death certificates submitted to the National Center for Health Statistics by all 50 states and DC. Denominator data obtained from the Current Population Survey. Data collection from 1992 to 2024 are relevant to our study. Data collection of the multiple causes of death through death certificates for National Vital Statistics began in 1968 and the process does not stop.
Timing	Annual cross-sections of deaths occurring between 1992 and 2024.
Data exclusions	Among non-Hispanic decedents aged 30-79, we exclude 2969754 deaths with missing education information, 1992 -2024.
Non-participation	NA. No participant dropped out or declined to participate because this study looks at death data only, and people cannot drop out from having deceased.
Randomization	NA — observational study; no intervention or allocation.

Reporting for specific materials, systems and methods

We require information from authors about some types of materials, experimental systems and methods used in many studies. Here, indicate whether each material, system or method listed is relevant to your study. If you are not sure if a list item applies to your research, read the appropriate section before selecting a response.

Materials & experimental systems

n/a	Involved in the study
☒	☐ Antibodies
☒	☐ Eukaryotic cell lines
☒	☐ Palaeontology and archaeology
☒	☐ Animals and other organisms
☒	☐ Clinical data
☒	☐ Dual use research of concern
☒	☐ Plants

Methods

n/a	Involved in the study
☒	☐ ChIP-seq
☒	☐ Flow cytometry
☒	☐ MRI-based neuroimaging

Plants

Seed stocks	N/A
Novel plant genotypes	N/A
Authentication	N/A
