# Supplementary material for: Smoking, education level and excess premature deaths in the USA
Source: Nat Health. 2026 Jul 10;1(11):1235–46. doi: 10.1038/s44360-026-00153-7 (PMC13727667; doi:10.1038/s44360-026-00153-7)
Supplement: Supplementary file 3 [file 44360_2026_153_MOESM3_ESM.pdf]

nature portfolio

Peer Review File

Smoking, education level and excess premature deaths in the USA

Corresponding Author: Dr Prabhat Jha

This manuscript has been previously reviewed at another journal. This document only contains information relating to versions considered at Nature Health.

Parts of this Peer Review File have been redacted as indicated to remove third-party material.

This file contains all reviewer reports in order by version, followed by all author rebuttals in order by version.

Version 0:

Reviewer comments:

Reviewer #1

(Remarks to the Author)
The main contribution of this paper is its assessment of smoking-related deaths as the single most important contributor to the mortality increase for low-educated adult Whites in the United States in the period 1989-2019 and to the growing education gradient in survival in that same group. Though its main findings could be of interest to the majority of the journal's national readership, I feel that it is not quite ready for publication given the issues listed below.

Introduction and background section
• The paper needs some more solid theoretical background on the known relationships between smoking, education, and mortality that should lead to clearly stated hypotheses. What were the authors expecting to find and why, given what is already known about the associations between mortality, education and race/ethnicity during the period of interest?
• Three essential papers on mortality patterns by smoking, race/ethnicity, and education are not mentioned as they should be, i.e.,
Jemal, A., Thun, M. J., Ward, E. E., Henley, S. J., Cokkinides, V. E., & Murray, T. E. (2008). Mortality from leading causes by education and race in the United States, 2001. American journal of preventive medicine, 34(1), 1-8.
Leive, A. A., & Ruhm, C. J. (2022). Education gradients in mortality trends by gender and race. Journal of Human Capital, 16(1), 47-72.
Montez, J. K., Hummer, R. A., Hayward, M. D., Woo, H., & Rogers, R. G. (2011). Trends in the educational gradient of US adult mortality from 1986 through 2006 by race, gender, and age group. Research on aging, 33(2), 145-171.
• In addition, more context is needed on historical change in smoking prevalence across cohorts and on patterns of smoking cessation by sex, race/ethnicity, and level of education, which should also help motivate the study and hypotheses. This information is essential to understand the results of the study.
• The authors underline "three key features of American society" but it is not clear to me why they selected these three features. There many more aspects of American society that might be more relevant (e.g., the large income inequalities as they relate to education). In particular, I do not understand why the lack of health insurance (mentioned here but also a couple more times throughout the paper without further explanations) is relevant here (how would that explain the education gradient in smoking-attributable mortality?)

Data and methods
• I had to read the Methods section before making sense of the Results. I would thus move this section up front, just after the introduction.
• This section should start with a clear description of the data used for the analysis. As the paper stands, I do not understand what were the sources of information for the study. The authors mention both the Current Population Surveys (CPS) and the National Death Index (NDI). Did the authors request linkage between the CPS samples (for all years 1989 to 2019) and the NDI? If that is the case, then the data refer to a random representative sample of the total US population so the authors should indicate the confidence intervals for all their results given that the CPS sample is fairly small (especially after disaggregating by sex, education level, race/ethnicity, and causes of death). If they used them independently (aggregating

the death counts by sex, race/ethnicity, level of education and cause of death for numerators of the rates and the CPS populations for the same variables and using the survey weights for the denominator), I assume that their mortality data do not come from the NDI but from the Mortality Multiple Cause Files produced and distributed by the NCHS at CDC.
• If they proceeded as described just above, then there are potential issues of numerator/denominator discrepancies given that each comes from a different source of information and this should be discussed in the paper limitations since the classification by level of education as well as race/ethnicity might have been different in the death certificate data (reported by the next of kin) and in the CPS (potentially reported by the person him/her-self).
• More generally, why not use the National Health Interview Survey Mortality-Linked files as all other studies on this issue have done (from which mortality rates can be calculated with numerators and denominators extracted from the same source, thus limiting numerator/denominator biases)? What are the advantages of using a combination of NDI and CPS data?
• I do not understand the sentence (lines 306-207) “The National Death Index includes automated methods to correct for occasional mistakes in death certification, such as confusing mode with underlying cause of death”; What types of mistakes are the authors referring to? What is “the mode” on a death certificate? Since they mention the underlying cause of death, they should provide the WHO definition and explain that the death certificate includes all the causes that have contributed to death, the underlying cause representing typically the "initial" cause of death (that is, the cause that initiated the sequence of events leading to death), which is also the cause reported by the NCHS in its publication on mortality by cause of death.
• Why restrict the analysis to Whites? Blacks have higher smoking rates and larger education disparities in smoking than Whites (Table 1 shows values for “non-white” thus suggesting that the calculations have already been carried out for other racial/ethnic groups). For instance, the authors emphasize that nearly three-quarters of low-education 30-year-old Whites would die before age 80” as a motivation to study this population group but the proportion is even higher among low-educated 30-year-old Blacks and the educational gradient is larger for Blacks than for Whites.
• The categorization of deaths needs to be justified. Why the emphasis on “accidental falls” (Extended Data Table 1) for instance (especially given that the analysis does not include people aged 80 years and above, which is where most of the mortality by fall occurs). Why not use or discuss other cause-of-death categories?
• The definition of education categories also needs to be motivated (why less than high-school, high-school and at least 1+ years of college rather than, for instance, high-school and lower, some college, completed college)? Or 4 rather 3 categories?
• Regarding the estimation of excess all-cause mortality based on the average annual rate of reduction in mortality of the high-education group, it is unclear what the reference is. Is it the average rate of decline between 1989-1991 and 2017-2019? Only the beginning period is indicated (1989-1991) here (line 320) while lines 76-77 indicates that the reference is “the average annual rate of mortality reduction in the high-education group from 1989 to 2019” (which should then be more accurately changed into “from 1989-1991 to 2017-2019”). Also, it would be more consistent to use the same periodicity throughout the paper (also use a 3-year moving average on Figures 1-4 or use a 2-year period for the reference – change in mortality for the highly educated from 1989-1990 to 2018-2019).
• As for the mortality indicator itself, as a demographer, I find it to be a bit messy and difficult to interpret directly as it is not exactly a hazard nor a probability of dying. The advantage of presenting the hazard itself (the Rp in the formula described in the "Statistical methods" section) is that, because hazards are additive, the ratio of the hazards is very close to the odds ratios with which people in the public health field are familiar with. The advantage of the probabilities is that they are either to understand intuitively (the average probability to have died by the end of the age interval for someone alive at the beginning of the interval). The probability is represented by
1-(1/exp(risk of death)) with the risk of death calculated as indicated by the authors.

Results
• Mortality divergence by education level appears to have been much larger for women than for men in the age group 30-64 years (with average annual increases in the mortality rate of 4% for women versus 3% for men in the low education group while they declined by 0.3% for women versus 1% for men), suggesting a larger expansion of the education gradient in mortality among women than men. Calls for some explanation (these values are from the note under Figure 1 but would deserve to be included in the text instead).
• Given the focus clearly stated from the beginning on adults above the age of 30 years, I would just drop the section on “Mortality trends at ages 15-29 years”. The focus is well justified given that smoking takes time to kill, thus mortality trends for this age group are irrelevant to the issue of the burden of smoking-related mortality on the education gradient in survival. For the same reason, I would also drop Table 5 and Figures 1 and 2 in the Supplemental materials. It would be more interesting to present results for other races than for the 15-29 years age group.
• It would be useful to distinguish between the biological pathways of influence of smoking on mortality (on Figure 5 and in the discussion). Both leading causes of death (cancer and cardiovascular diseases) are strongly associated with smoking, but with very different timing. There is a large body of literature demonstrating that there is a 30 year-lag between smoking uptake and cancer mortality and that people who stop smoking still experience excess mortality compared to the never-smokers. By contrast, when people stop smoking, not only is the effect on their mortality risks from cardiovascular diseases immediate but after a few years, their risks become similar to those of the never-smokers.
• The authors emphasize that “this stagnation [of mortality in low-educated White people] has contributed to the perception of an epidemic of chronic diseases, whereas the reality is more nuanced” (lines 206-207), presumably based on their finding that “smoking is the single most significant contributor to the stagnation of survival of the past three decades” (lines 209-2010). However, smoking does kill primarily through the two main chronic diseases, cancer and cardiovascular diseases so that would justify the statement about an epidemic of chronic diseases.
Discussion
• This is the section that requires the most work.
• There should absolutely be more discussion of compositional and selection effects because the education distribution of people who are 65 to 79 years in 1989 is vastly different from that of people who are 30 to 64 years old in 2019 (in 1989, people aged 30-64 years were born in 1925-1959 and those aged 65-79 years were born in 1910-1924; in 2019, those aged

30-64 were born in 1955-1989 and those aged 65-79 years were born in 1940-1954). People with low levels of education in the 1910-1924 cohorts were very different than those in the 1955-1989 cohorts and this would have been enough to explain some of the results. In addition, the 1910-1924 cohorts are those which have exhibited the highest prevalence of smoking (they smoked more than both the cohorts born before 1910 and the cohorts born after 1924), with large differences by race/ethnicity (Whites smoked more in the early cohorts and Blacks in the most recent cohorts). Again, these differential patterns need to be emphasized and discussed in relation to the study findings.

• More generally, I believe that the discussion could be much improved by explaining thoroughly the potential role of composition effects (more selection mediated through education as time goes on – as mentioned on lines 268-269 but without explaining the implications) versus rate effects. Similarly, the authors should try to disentangle cohort (i.e., the large differences in smoking prevalence between the 1910-1924 cohort and the 1955-1989 cohort) and period effects (smoking cessation campaigns of the 1970s that encouraged people to stop smoking and discouraged young adults to take up smoking) with a strong education gradient (though initially, more smoking among the more educated, especially among women)

• This section should also provide more discussion of the study results versus those from other studies on education, race/ethnicity and mortality trends covering all or parts of the same time period and age groups (in particular those by Leive and Ruhm, 2022; Jemal et al., 2008; Montez et al., 2011 previously mentioned but also others with specific results on the same issue). For instance, Leive and Ruhm found that deaths from respiratory diseases have contributed at least as much as deaths from suicides and chronic live diseases to the increased mortality of low-educated Whites (especially women) and to the educational gradient in mortality. In addition, several authors have found that drug overdoses were the main culprit in the increasing mortality of the years 2014-2017 and, more generally, to the lack of improvement among low-educated adults in the US after 2010, which is in direct opposition to the authors' statement that "smoking remained the dominant factor and these ages and played an even larger role in excess deaths among lower-education groups at ages 65-79 [after 2010]" (lines 217-219). Why the difference with the current study?

• A more minor comment is that I do not understand the reference to "childhood vaccine schedule" (line 230) as an explanation for stagnating mortality among low-educated White people (do the authors mean "childhood vaccination rates"?).

• By contrast with what the authors claim ("exposures that would explain the crises in US survival would need to be concentrated in lowest education or income groups, which does not appear to be the case for most exposures" – lines 240-242), there is an increasing body of literature demonstrating how poor and minority populations are concentrated in communities suffering higher rates of air, soil, and water pollution than highly-educated White people.

• When the authors argue that attention to more contextual factors as explaining the education gradient in mortality is a distraction from the "biggest avoidable risks, most notably smoking, opioids and alcohol and poisonings" (lines 246-1247), they should also mention that it is easier, from a policy perspective, for the government to change the tax structure or to increase regulations that could affect air, soil and water pollution than to modify individual behavior (especially if those behavior have to do with people's objective socio-economic conditions and distress).

• What kind of "subtle advantages in effective health insurance coverage" could have benefited the low-educated population (lines 270-271)?

Tables and Figures

• The notes under Table 1 are unclear ("Rp is the 5-year age-specific all-cause mortality rate for the 15 years" => what 15 years? ; "By comparison the population in millions of low/high in 1989 was 18.8 and 41.3, respectively. By 2019, it was 4.3 and 84.1, respectively" => "low/high" what? Low education versus high education?)

• Use log scale on the y-axis for figures 1-4 since the main focus is change over time

• In the extended Tables 1 and 2, what is labeled "vascular" diseases should be "cardio-vascular" diseases as not all diseases in the ICD chapter I00-I99 are related to blood vessels (some heart diseases affect the heart independently from blood vessels).

(Remarks on code availability)

Reviewer #2

(Remarks to the Author)

Thank you for the opportunity to review the paper. The authors investigate the stagnation (and in some cases reversal) of working-age and older mortality patterns in the United States, particularly among less-educated non-Hispanic whites. The decompose these changes across education groups by smoking-attributable and smoking non-attributable mortality, using the method by Peto et al to map causes of death onto this risk factor. Their core findings are of large increases in mortality for individuals with less than high school education, of which a substantial fraction is attributable to smoking.

This is an interesting research question and I appreciate the hard work that went into this study. It is plausible that smoking may explain an important portion of the yawning gaps by education in working-age mortality. However, I do have some concerns:

1. The "low" education group is an increasingly selected group over time, particularly over the last 30 years. The type of individual with less than an HS degree today is very different than in the 1990s, and is likely worse off on many dimensions. (My guess is that the fraction of the sample reporting "low" education has decreased over the study period considerably). Novosad and colleagues (AEJ Applied: https://www.aeaweb.org/articles?id=10.1257/app.20190297) consider this form of selection, and focus instead on percentiles of years of education (which are constant over time), using a partial identification approach to bound estimates. The Novosad paper suggests that the present estimates of increasing mortality in the low education group are almost certainly too large. I would love to see the selection corrected versions as a robustness check, at

least.

2. Keeping #1 in mind, for the high education group, who not separate into "some college" (13-15) and four year college and above (16+)? The latter group would be in direct conversation with work by Case, Deaton, Schwandt, Currie, Hayward and many others.

3. The counterfactual standard here is the "high education" group. However, mortality has slowed in this group, too. I wonder if an international counterfactual might be better for this exercise (e.g., https://pubmed.ncbi.nlm.nih.gov/37303714/). E Table 6 addresses this to some extent, but other data suggest that even the best off middle-aged Americans do worse than the poorest Europeans (https://www.nejm.org/doi/abs/10.1056/NEJMsa2408259)

4. The authors acknowledge that smoking rates have gone down for lower educated Americans, just less so than other groups. That could explain diverging rates by education. But how does that square with an *increase* in mortality in this group? I might be misunderstanding, so apologies if so, but the only way this could occur is if smoking is deadlier today than it was previously I.e., we have P(smoking)*P(mortality|smoking), we know P(smoking) is going down, so P(mortality|smoking) must be going up. Has that been shown?

5. There may be other methods to estimate smoking attributable fractions besides Doll and colleagues, e.g.: https://pmc.ncbi.nlm.nih.gov/articles/PMC6219821/ or https://pmc.ncbi.nlm.nih.gov/articles/PMC6351587/. It would be interesting to assess the robustness of the findings to alternate methods.

(Remarks on code availability)
I read the stata code specifically to see how smoking attributable mortality was calculated.

Reviewer #3

(Remarks to the Author)
The study examines education-based gaps in all-cause and cause-specific mortality in the United States from 1989 to 2023. The analysis relies on death certificates (numerator) and population estimates (denominator) from the Current Population Survey. The findings show that the mortality gap by educational attainment level grew among whites, both men and women, throughout the study period. Growing mortality gaps were observed across multiple causes of death, most notably due to cancer and opioid poisoning, cirrhosis, and external causes. These trends resulted in excess deaths among lower education groups.

Overall, the findings in this study are far from novel and have been documented in the research literature countless times, often using better methods and more nuanced analyses. There are also some methodological limitations that have been raised by previous reviewers and were not adequately addressed by the authors.

Major comments:

1. The educational gradient in mortality is well established; so are gaps in specific causes of death (Miech et al. 2011), the contribution of smoking to the overall mortality gap (Ho & Fenelon 2015), and the contribution of education-based inequalities to excess deaths or life years lost (Sasson & Hayward 2019); critique of the “deaths of despair” hypotheses is also fairly old by now (Masters et al. 2017; Ruhm 2022). Thus, the study’s key contribution is unclear with respect to the extant literature. Regrettably, the authors do not engage with much of this literature and do not attempt to highlight what is new about their analysis, findings, or conclusions.

2. Responses to the previous reviewers were not particularly effective in answering key concerns about the methods or data. Missing data are poorly handled. Education misreporting in unlinked data is also a reason for concern, though I do not believe that it completely invalidates the findings. Unfortunately, the authors opted not to engage with these issues directly (e.g. conduct sensitivity analyses, benchmark against other sources) and merely acknowledged them in the limitations. For example, they reply that “mortality-linked surveys do not span nearly as long as three decades, we opted to use the unlinked data for this analysis.” However, the NHIS linked mortality files date back the 1980s and permit precisely this type of analysis, at the very least for all-cause mortality. The estimates could also be compared with other surveys in later periods.

Minor comments:

3. The third paragraph of the introduction states that “a proper understanding of the stagnation in US adult survival requires consideration of three key features of American society,” namely racial and ethnic disparities in mortality, the prominence of smoking behavior in driving mortality patterns and trends, and inequalities in access to healthcare. While all of those factors are indeed important, no evidence or reasoning is given to support the claim that they should be singled out from many other demographic, behavioral, or socio-political factors. Furthermore, the paper goes on to examine only one of those factors (smoking). If racial disparities are so important for understanding the US mortality landscape, why does the study ignore them altogether and focuses only on non-Hispanic whites? Health insurance coverage is also completely absent from the data and analysis, so why mention it so prominently in the introduction?

4. Prior research has shown that the most significant educational cutoff is having a college degree (Case & Deaton 2021; Sasson & Hayward 2019). However, the present study categorized educational attainment as less than high-school (<11 years of schooling), high-school (12 years), and higher (13+ years). What was the main reasoning behind this

categorization?

5. The paper includes a number of inaccuracies and odd phrasings. For example, it is claimed that US mortality started stagnating around 1980. That is simply not true. Stagnation started around 2010, which is well documented and studied. Another example is “stagnating survival has significant political repercussions” (p.1 second paragraph). Given the rest of the paragraph, I believe the authors meant that stagnant mortality has social and political causes, rather than consequences.

References

Case, A., & Deaton, A. (2021). Life expectancy in adulthood is falling for those without a BA degree, but as educational gaps have widened, racial gaps have narrowed. Proceedings of the National Academy of Sciences, 118(11), e2024777118.

Ho, J. Y., & Fenelon, A. (2015). The contribution of smoking to educational gradients in US life expectancy. Journal of Health and Social Behavior, 56(3), 307-322.

Masters, R. K., Tilstra, A. M., & Simon, D. H. (2017). Mortality from suicide, chronic liver disease, and drug poisonings among middle-aged US white men and women, 1980–2013. Biodemography and Social Biology, 63(1), 31-37.

Miech, R., Pampel, F., Kim, J., & Rogers, R. G. (2011). The enduring association between education and mortality: the role of widening and narrowing disparities. American Sociological Review, 76(6), 913-934.

Ruhm, C. J. (2022). Living and dying in America: An essay on deaths of despair and the future of capitalism. Journal of Economic Literature, 60(4), 1159-1187.

Sasson, I., & Hayward, M. D. (2019). Association between educational attainment and causes of death among white and black US adults, 2010-2017. JAMA, 322(8), 756-763.

(Remarks on code availability)

Version 1:

Reviewer comments:

Reviewer #2

(Remarks to the Author)
The authors have responded sufficiently to my concerns. I also appreciate their responses to the other referees.

(Remarks on code availability)

Reviewer #3

(Remarks to the Author)
The revised version is considerably improved. The key contribution is more clearly articulated, and the methods are introduced in greater detail. The authors also present a commendable sensitivity analysis comparing three methods to attributed deaths to smoking. However, the manuscript still has notable limitations that potentially undermine its key argument.

1. As another reviewer previously commented, the authors use multiple indicators of mortality (risks, rates, excess deaths) interchangeably throughout the manuscript. This practice not only makes the paper difficult to follow, but also potentially undermines some of its key arguments. For example, the authors note in the discussion that “smoking accounts for over three-fifths of the trends in educational inequalities in mortality in the US.” It appears that this statement is based on Figure 5, which has no reference in the text, and portrays the absolute number of excess deaths attributed to smoking among low and middle-educated Americans. However, the absolute number of excess deaths also depends on the population composition, and not only on disparities in mortality rates. Thus, arguments and hypotheses should be stated more accurately throughout the paper and particularly whether they relate to the actual US population, some hypothetical construct (e.g., age-standardized or stationary population), or the risk of dying from an individual’s standpoint.

2. Compositional changes are largely ignored, despite being critical for understanding mortality inequalities (see van Raalte et al. 2025). It is also unclear how these concerns are addressed by the Peto method as the authors assert in their rebuttal. Related to this issue, it is not entirely clear if step 1 of the Peto method entails age standardization as the age intervals used are fairly large (e.g., 30-64), which could also introduce biases in the numerator and denominator across education groups.

3. Figures 3 & 4 do not seem to add up. For example, among men aged 30-64 the mortality rate attributed to smoking has more than doubled from 300 to 700 deaths per 100,000 per year (Figure 3). However, the breakdown to major smoking-attributable causes in Figure 4 scarcely add up to this total. What is missing?

4. Missing data: the authors excluded 1.5 million deaths with unknown educational attainment level. Although it is a relatively small proportion of all deaths observed during the study period (33 million), they may be concentrated in specific years and education groups. These cases should be treated with greater care.

5. It would help to include a detailed table with descriptive statistics showing the number of deaths in each education group/year.

References

van Raalte, A., Vierboom, Y., & Martikainen, P. (2025). Revisiting within-cohort compositional change to understand mortality inequalities. Vienna Yearbook of Population Research, 23(1), 43-57.

(Remarks on code availability)
No code was provided.

Open Access This Peer Review File is licensed under a Creative Commons Attribution 4.0 International License, which permits use, sharing, adaptation, distribution and reproduction in any medium or format, as long as you give appropriate credit to the original author(s) and the source, provide a link to the Creative Commons license, and indicate if changes were made.
In cases where reviewers are anonymous, credit should be given to 'Anonymous Referee' and the source.
The images or other third party material in this Peer Review File are included in the article's Creative Commons license, unless indicated otherwise in a credit line to the material. If material is not included in the article's Creative Commons license and your intended use is not permitted by statutory regulation or exceeds the permitted use, you will need to obtain permission directly from the copyright holder.
To view a copy of this license, visit https://creativecommons.org/licenses/by/4.0/

Response to Reviewers:

We thank the editors and reviewers for their careful reading of our manuscript and their constructive feedback and apologize for the delay in replying (partly as one of us was starting a new position in Oxford). We have revised the manuscript to address the concerns raised, expanded the study to cover deaths until 2024, added new analyses including stratifying deaths attributable and not attributable to smoking by education group as well as trends in current and never smoking, obesity and alcohol use by education group. We realize that our replies may not address the points raised by reviewer 3, but disagree with his/her assertion that we have not addressed earlier review comments.

Using quantitative analysis, our makes points that are qualitatively important, namely that (a) the stagnation is concentrated in the low education group and that (b) smoking is overwhelmingly the cause of the differential mortality by education group. Subtleties of analysis, such as some of the thoughtful comments from reviewers, would be great to address in an ideal world with sufficiently large, representative prospective studies. But they would not likely alter our important qualitative findings.

Editor comments and responses in separate document

Reviewer #1

Introduction and background section
Comment 4: The paper needs some more solid theoretical background on the known relationships between smoking, education, and mortality that should lead to clearly stated hypotheses. What were the authors expecting to find and why, given what is already known about the associations between mortality, education and race/ethnicity during the period of interest?

Response 4: The introduction now frames our hypotheses explicitly: that smoking is the primary contributor to widening educational mortality gradients among Whites. Understanding the contributions of smoking needs proper understanding of other causes- such as opioids, as well as those from cirrhosis, suicides and external causes. While reliable methods to assign obesity as cause of death to various education strata do not exist, we have added Appendix material on the trends in key risk factors, including obesity and binge alcohol. We also explain that documenting reliably the contribution or smoking to mortality in various educational strata is challenging, but the benefit of the lung-cancer indexed (Peto) method is that it combines, implicitly, age, period and cohort effects.

More precisely, the Peto method is meant to approximate population cumulative exposure to smoking in settings where reliable survey data on lifetime smoking patterns are unavailable. Lung cancer deaths reflect long, lagged smoking histories — typically cumulative exposure 20–30 years prior (but less for younger ages). This means observed lung cancer death rates incorporate cohort effects (when birth cohorts began smoking, at what intensity), as well as period effects (policy shifts, tobacco availability, cessation campaigns).

The Peto model does not explicitly model age, period, or cohort effects. Instead, it estimates these as follows:

- **Age effects: accounted for indirectly because the smoking impact ratio is computed in age groups (e.g., 35–69, or 5-year bands). This means older age groups reflect the longer cumulative exposure and higher risk of lung cancer mortality.**
- **Cohort effects: absorbed indirectly through the lung cancer death rates in each age group. If a cohort smoked more heavily earlier in life, their excess lung cancer deaths show up in the numerator.**
- **Period effects: also implicit, because the model uses observed lung cancer death rates from a given calendar period. If lung cancer risk changes (say due to reduced tar content), this shifts the measured lung cancer rates.**

So, our analysis indexes cumulative smoking exposure to observed lung cancer mortality rather than attempting to separately estimate age, period, cohort components.

We then compare smoking to other causes of death that have been defined as "diseases of despair" such as opioids (noting that some deaths from opioids might show up in other ICD-10 certified medical causes, such as suicide), liver cirrhosis, suicide, and external causes.

Comment 5: Three essential papers on mortality patterns by smoking, race/ethnicity, and education are not mentioned as they should be, i.e.,
Jemal, A., Thun, M. J., Ward, E. E., Henley, S. J., Cokkinides, V. E., & Murray, T. E. (2008). Mortality from leading causes by education and race in the United States, 2001. American journal of preventive medicine, 34(1), 1-8.
Leive, A. A., & Ruhm, C. J. (2022). Education gradients in mortality trends by gender and race. Journal of Human Capital, 16(1), 47-72.
Montez, J. K., Hummer, R. A., Hayward, M. D., Woo, H., & Rogers, R. G. (2011). Trends in the educational gradient of US adult mortality from 1986 through 2006 by race, gender, and age group. Research on aging, 33(2), 145-171.

Response 5: Thank you. We now cite and build on the three key studies suggested.

Comment 6: In addition, more context is needed on historical change in smoking prevalence across cohorts and on patterns of smoking cessation by sex, race/ethnicity, and level of education, which should also help motivate the study and hypotheses. This information is essential to understand the results of the study.

Response 6: We emphasize that by using the lung-cancer index method, we need not rely on changing smoking incidence. Mortality effects using lung cancer overcome the limitations of smoking prevalence surveys and incorporate also change in type of smoking (see Response 4).

We emphasize that while there have been important declines in overall prevalence of smoking, the declines have been slower in older and lower education age groups. For example, in the National Survey of Drug Use: Current smoking decreased significantly during 2002–2019 (27.4% to 18.2%). But the slowest rates of decline in the prevalence of current cigarette smoking were among adults aged 50 years or older (17.3% to 14.7%; relative percentage change [RPC] = −15.0%) and among those who had less than a high school diploma (35.2% to 26.7%; RPC = −24.1%). By contrast, the largest declines in the prevalence of current cigarette smoking by education are among the most educated group (respondents with a college education or more, 14.5% to 8.1%; RPC = −44.1%); and among adults aged 18 to 25 years (40.8% to 17.8%; RPC = −56.4%.)[6,7]

These data are now summarized in the introduction.

Comment 7: The authors underline "three key features of American society" but it is not clear to me why they selected these three features. There many more aspects of American society that might be more relevant (e.g., the large income inequalities as they relate to education). In particular, I do not understand why the lack of health insurance (mentioned here but also a couple more times throughout the paper without further explanations) is relevant here (how would that explain the education gradient in smoking-attributable mortality?)

Response 7: Thank you. We have dropped for clarity so the above three features are now moot.

Data and methods

Comment 8: I had to read the Methods section before making sense of the Results. I would thus move this section up front, just after the introduction.

Response 8: We followed Nature's format which puts methods at the end of the manuscript. The introduction does however summarize the methods.

Comment 9: This section should start with a clear description of the data used for the analysis. As the paper stands, I do not understand what were the sources of information for the study. The authors mention both the Current Population Surveys (CPS) and the National Death Index (NDI). Did the authors request linkage between the CPS samples (for all years 1989 to 2019) and the NDI? If that is the case, then the data refer to a random representative sample of the total US population so the authors should indicate the confidence intervals for all their results given that the CPS sample is fairly small (especially after disaggregating by sex, education level, race/ethnicity, and causes of death). If they used them independently (aggregating the death counts by sex, race/ethnicity, level of education and cause of death for numerators of the rates and the CPS populations for the same variables and using the survey weights for the denominator), I assume that their mortality data do not come from the NDI but from the Mortality Multiple Cause Files produced and distributed by the NCHS at CDC.

Response 9: We thank the reviewer as his/her not understanding our methods suggests we need to simplify and explain them better. We now explain that we used only the age and sex-specific proportions of CPS population by education group to assign to national totals. These are the denominators. The numerators are national mortality data. Mortality data are from the Mortality Multiple Cause Files. We do not link CPS samples to the NDI.

Comment 10: If they proceeded as described just above, then there are potential issues of numerator/denominator discrepancies given that each comes from a different source of information and this should be discussed in the paper limitations since the classification by level of education as well as race/ethnicity might have been different in the death certificate data (reported by the next of kin) and in the CPS (potentially reported by the person him/her-self).

Response 10: We thank the reviewer for pointing this out. We have added a reference to support the point that post-mortem proxy reporting of education tends to overreport less than high school education as high school education. Presumably, the self-reported CPS education data does not have this bias. This would tend to underestimate the death rates from lung cancer and other disease categories in the lowest education group. However, there is no reason to think that such biases have changed over time. In the methods section, we have added text to cover this point in detail, and included citations to changes in misclassification of education over time.

Comment 11: More generally, why not use the National Health Interview Survey Mortality-Linked files as all other studies on this issue have done (from which mortality rates can be calculated with numerators and denominators extracted from the same source, thus limiting numerator/denominator biases)? What are the advantages of using a combination of NDI and CPS data?

Response 11: We opted not to use the NHIS-linked mortality files (NHIS-LMF) because it is too small to examine the specific racial, sex and age groups of interest for cause specific mortality. We needed more granular detail on the causes of death than the variables provided by the NHIS-LMF. In NHIS-LMF, the causes of death are summarized in the variable UCOD_113 which represents 113 causes of deaths based on ICD-10 codes and their ICD-9 equivalents. Although this variable is largely adequate for most causes of deaths in our study, we needed the T codes from the Multiple Causes of Death Mortality Data to properly identify opioid-related death. Furthermore, we prefer using comprehensive population-level data whenever possible, as opposed to relying on sample weights for nationally representative surveys.

Comment 12: I do not understand the sentence (lines 306-207) “The National Death Index includes automated methods to correct for occasional mistakes in death certification, such as confusing mode with underlying cause of death”; What types of mistakes are the authors referring to? What is “the mode” on a death certificate? Since they mention the underlying cause of death, they should provide the WHO definition and explain that the death certificate includes all the causes that have contributed to death, the underlying cause representing typically the

"initial" cause of death (that is, the cause that initiated the sequence of events leading to death), which is also the cause reported by the NCHS in its publication on mortality by cause of death.

Response 12: We have revised this section for clarity. Mode of death refers to the" immediate" cause of death (like cardiorespiratory arrest). "Underlying" cause of death as a concept is well described and understood in the literature.

Comment 13: Why restrict the analysis to Whites? Blacks have higher smoking rates and larger education disparities in smoking than Whites (Table 1 shows values for "non-white" thus suggesting that the calculations have already been carried out for other racial/ethnic groups). For instance, the authors emphasize that nearly three-quarters of low-education 30-year-old Whites would die before age 80" as a motivation to study this population group but the proportion is even higher among low-educated 30-year-old Blacks and the educational gradient is larger for Blacks than for Whites.

Response 13: See Reply 1. In addition, the Leive and Ruhm, 2022 paper notes that mortality trends in different races are heterogenous, with the largest drop in mortality occurring in US Blacks.

Comment 14: The categorization of deaths needs to be justified. Why the emphasis on "accidental falls" (Extended Data Table 1) for instance (especially given that the analysis does not include people aged 80 years and above, which is where most of the mortality by fall occurs). Why not use or discuss other cause-of-death categories?

Response 14: We have dropped the falls analyses. Our focus is on avoidable mortality, which is larger for below 80 than above 80 years.

Comment 15: The definition of education categories also needs to be motivated (why less than high-school, high-school and at least 1+ years of college rather than, for instance, high-school and lower, some college, completed college)? Or 4 rather 3 categories?

Response 15: See Response 3 and Reply Figure 1 where we have addressed these points.

As noted, we've chosen to retain our original education groupings in the paper as it is more conservative and practical for public health targeting (say smoking cessation programs targeted for those with high school or less education).

Comment 16: Regarding the estimation of excess all-cause mortality based on the average annual rate of reduction in mortality of the high-education group, it is unclear what the reference is. Is it the average rate of decline between 1989-1991 and 2017-2019? Only the beginning period is indicated (1989-1991) here (line 320) while lines 76-77 indicates that the reference is "the average annual rate of mortality reduction in the high-education group from 1989 to 2019" (which should then be more accurately changed into "from 1989-1991 to 2017-2019"). Also, it would be more consistent to use the same periodicity throughout the paper (also use a 3-year moving average on Figures 1-4 or use a 2-year period for the reference – change in mortality for the highly educated from 1989-1990 to 2018-2019).

Response 16: We have edited this section for more clarity. We have excluded the early years due to higher proportion of missing data. The reference point for the average rate of decline is the now mean of 1992 and 1993.

Comment 17: As for the mortality indicator itself, as a demographer, I find it to be a bit messy and difficult to interpret directly as it is not exactly a hazard nor a probability of dying. The advantage of presenting the hazard itself (the Rp in the formula described in the "Statistical methods" section) is that, because hazards are additive, the ratio of the hazards is very close to the odds ratios with which people in the public health field are familiar with. The advantage of the probabilities is that they are easier to understand intuitively (the average probability

to have died by the end of the age interval for someone alive at the beginning of the interval). The probability is represented by 1-(1/exp(risk of death)) with the risk of death calculated as indicated by the authors.

Response 17: We are reporting risk simply. As deaths are rare events, the rate, hazard and probability are nearly identical.

In our search of articles published by Nature and its family of journals, risks (Jin 2020), hazards (Anzalone 2025) and rates (Chu 2025) have all been reported in various studies. [10–12]

Jin, J., Agarwala, N., Kundu, P. *et al.* Individual and community-level risk for COVID-19 mortality in the United States. *Nat Med* 27, 264–269 (2021). https://doi.org/10.1038/s41591-020-01191-8; Anzalone, A.J., Vest, M.T., Schissel, M.E. *et al.* Higher mortality following SARS-CoV-2 infection in rural versus urban dwellers persists for two years post-infection. *Nat Commun* 16, 8933 (2025). https://doi.org/10.1038/s41467-025-63944-8; Chu, L., Warren, J.L., Spatz, E.S. *et al.* Floods and cause-specific mortality in the United States applying a triply robust approach. *Nat Commun* 16, 2853 (2025). https://doi.org/10.1038/s41467-025-58236-0

That being said, we agree with the reviewer that presentation of risks if helpful to the individual information, so have added the risk of death to the key figures as a secondary Y-axis, complementing the age-standardized death rates on the primary Y-axis.

Results

Comment 18: Mortality divergence by education level appears to have been much larger for women than for men in the age group 30-64 years (with average annual increases in the mortality rate of 4% for women versus 3% for men in the low education group while they declined by 0.3% for women versus 1% for men), suggesting a larger expansion of the education gradient in mortality among women than men. Calls for some explanation (these values are from the note under Figure 1 but would deserve to be included in the text instead).

Response 18: We thank the reviewer for this point. We now highlight this result in the Results and expand the Discussion to consider gendered patterns in smoking. The smoking-attributable/not attributable stratified analysis by cause of death also adds important nuance to differences between women and men. We speculate that in particular increases in vascular disease mortality and COPD may play a role in the larger divergence by education among women than men.

Comment 19: Given the focus clearly stated from the beginning on adults above the age of 30 years, I would just drop the section on “Mortality trends at ages 15-29 years”. The focus is well justified given that smoking takes time to kill, thus mortality trends for this age group are irrelevant to the issue of the burden of smoking-related mortality on the education gradient in survival. For the same reason, I would also drop Table 5 and Figures 1 and 2 in the Supplemental materials. It would be more interesting to present results for other races than for the 15-29 years age group.

Response 19: We agree. We have removed ages 15-29 analyses completely.

Comment 20: It would be useful to distinguish between the biological pathways of influence of smoking on mortality (on Figure 5 and in the discussion). Both leading causes of death (cancer and cardiovascular diseases) are strongly associated with smoking, but with very different timing. There is a large body of literature demonstrating that there is a 30 year-lag between smoking uptake and cancer mortality and that people who stop smoking still experience excess mortality compared to the never-smokers. By contrast, when people stop smoking, not only is the effect on their mortality risks from cardiovascular diseases immediate but after a few years, their risks become similar to those of the never-smokers.

Response 20: See response 4, where we point out the benefit of the lung-cancer indexed method to provide a practical level of effective smoking exposure causing diseases other than lung cancer. We now have added appendix material examining the trends in the US NHIS studies aged 30-64 for their current and never

smoking. We discuss this possibility in the context of differences in Figures 3 and 4, showing the smoking/not smoking attributable deaths from various causes, in particular the marked increase in COPD mortality in the lower education groups. New text has been added to the results and the discussion, including the concluding paragraph.

Comment 21: The authors emphasize that "this stagnation [of mortality in low-educated White people] has contributed to the perception of an epidemic of chronic diseases, whereas the reality is more nuanced" (lines 206-207), presumably based on their finding that "smoking is the single most significant contributor to the stagnation of survival of the past three decades" (lines 209-2010). However, smoking does kill primarily through the two main chronic diseases, cancer and cardiovascular diseases so that would justify the statement about an epidemic of chronic diseases.

Response 21: We agree this could be more clearly written, and have accordingly revised.

Discussion

Comment 22: There should absolutely be more discussion of compositional and selection effects because the education distribution of people who are 65 to 79 years in 1989 is vastly different from that of people who are 30 to 64 years old in 2019 (in 1989, people aged 30-64 years were born in 1925-1959 and those aged 65-79 years were born in 1910-1924; in 2019, those aged 30-64 were born in 1955-1989 and those aged 65-79 years were born in 1940-1954). People with low levels of education in the 1910-1924 cohorts were very different than those in the 1955-1989 cohorts and this would have been enough to explain some of the results. In addition, the 1910-1924 cohorts are those which have exhibited the highest prevalence of smoking (they smoked more than both the cohorts born before 1910 and the cohorts born after 1924), with large differences by race/ethnicity (Whites smoked more in the early cohorts and Blacks in the most recent cohorts). Again, these differential patterns need to be emphasized and discussed in relation to the study findings.

Response 22: See Response 4, where we explain how the use of lung cancer indexed methods captures some of these cohort and period effects. Nonetheless, teasing out age-period and cohort analyses is always tricky, and particularly so when looking at different education groups. We have added references to Novosad *et al.* (2019)[15] and others. We also point out these issues in the limitation section.

Comment 23: More generally, I believe that the discussion could be much improved by explaining thoroughly the potential role of composition effects (more selection mediated through education as time goes on – as mentioned on lines 268-269 but without explaining the implications) versus rate effects. Similarly, the authors should try to disentangle cohort (i.e., the large differences in smoking prevalence between the 1910-1924 cohort and the 1955-1989 cohort) and period effects (smoking cessation campaigns of the 1970s that encouraged people to stop smoking and discouraged young adults to take up smoking) with a strong education gradient (though initially, more smoking among the more educated, especially among women).

Response 23: See Response to editors, and response 4. There are insufficient data on the specific patterns of smoking duration, intensity and amount and the types of cigarettes consumed (e.g., high or low tar) linked to cause-specific mortality by education and age/sex to enable the analyses proposed by the reviewer. Even if they were available, examination of differences by period and cohort are difficult to quantify reliably as they can be co-linear. Even it was possible to add these analyses, they would not alter our qualitative conclusions.

Comment 24: This section should also provide more discussion of the study results versus those from other studies on education, race/ethnicity and mortality trends covering all or parts of the same time period and age groups (in particular those by Leive and Ruhm, 2022; Jemal et al., 2008; Montez et al., 2011 previously mentioned but also others with specific results on the same issue). For instance, Leive and Ruhm found that deaths from respiratory diseases have contributed at least as much as deaths from suicides and chronic live diseases to the increased mortality of low-educated Whites (especially women) and to the educational gradient

in mortality. In addition, several authors have found that drug overdoses were the main culprit in the increasing mortality of the years 2014-2017 and, more generally, to the lack of improvement among low-educated adults in the US after 2010, which is in direct opposition to the authors' statement that "smoking remained the dominant factor and these ages and played an even larger role in excess deaths among lower-education groups at ages 65-79 [after 2010]" (lines 217-219). Why the difference with the current study?

Response 24: Ours is the first study, as far as we can determine, that has examined differences in education mortality over an approximate three-decade period and examine the many diseases that can be made more common by smoking. We now provide results stratified by smoking attributable/non attributable for the major causes. We compare the contribution of smoking attributable mortality to opioids and other diseases, to place our findings in context. The major conclusion is that smoking accounts for about half of the excess mortality observed in the lowest education groups compared to those with more than high school education, and that even in the 2010-19 period, when the contribution of opioids rose sharply, smoking still caused 45% of the excess deaths. These findings have not been documented previously.

Comment 25: A more minor comment is that I do not understand the reference to "childhood vaccine schedule" (line 230) as an explanation for stagnating mortality among low-educated White people (do the authors mean "childhood vaccination rates"?).

Response 25. We agree this is confusing and have dropped it.

Comment 26: By contrast with what the authors claim ("exposures that would explain the crises in US survival would need to be concentrated in lowest education or income groups, which does not appear to be the case for most exposures" – lines 240-242), there is an increasing body of literature demonstrating how poor and minority populations are concentrated in communities suffering higher rates of air, soil, and water pollution than highly-educated White people.

Response 26: We have edited this section for clarity to make the point that residual confounding of the association of smoking with various diseases (say by alcohol) is reasonably small. The stratified analyses of mortality attributable to smoking or not attributable to smoking enables a crude examination of other factors, such as obesity. Obesity is the most probable explanation for rising vascular mortality in non-smokers.

Comment 27: When the authors argue that attention to more contextual factors as explaining the education gradient in mortality is a distraction from the "biggest avoidable risks, most notably smoking, opioids and alcohol and poisonings" (lines 246-247), they should also mention that it is easier, from a policy perspective, for the government to change the tax structure or to increase regulations that could affect air, soil and water pollution than to modify individual behavior (especially if those behavior have to do with people's objective socio-economic conditions and distress).

Response 26: We agree and have edited this section to emphasize that further research in the social and environment exposures of the lowest education groups is needed. We believe that the reviewer would agree that the actionable determinants, such as smoking and opioid control need not wait for further research.

Comment 28: What kind of "subtle advantages in effective health insurance coverage" could have benefited the low-educated population (lines 270-271)?

Response 27: According to US Census data from 2021, 30% of those with less than HS level education lacked health insurance, and only 5% of those with BA+ were uninsured for age 25-64.[16] Forgoing care or medication due to cost isn't a myth. Having some insurance coverage could help the lower education group to seek preventive care or seek care when health conditions were less dire and more reversable. A full discussion of health insurance coverage is beyond the scope of this paper, but we have edited the above for clarity. See as a reference: Branch, B. 2023. Differences in Uninsured Rates by Race and Ethnicity Persist Even Among Those

With Higher Educational Attainment https://www.census.gov/library/stories/2023/03/education-and-racial-disparities-in-health-insurance-coverage.html

Tables and Figures

Comment 29: The notes under Table 1 are unclear ("Rp is the 5-year age-specific all-cause mortality rate for the 15 years" => what 15 years? ; "By comparison the population in millions of low/high in 1989 was 18.8 and 41.3, respectively. By 2019, it was 4.3 and 84.1, respectively" => "low/high" what? Low education versus high education?)

Response 29: We have edited the Table to improve clarity.

Comment 30: Use log scale on the y-axis for figures 1-4 since the main focus is change over time

Response 30: We politely disagree. Given that people die on an arithmetic and not a log scale, we prefer to keep our current scale.

Comments 31: In the extended Tables 1 and 2, what is labeled "vascular" diseases should be "cardio-vascular" diseases as not all diseases in the ICD chapter I00-I99 are related to blood vessels (some heart diseases affect the heart independently from blood vessels).

Response 31: Vascular is commonly used interchangeably with cardiovascular. But we have clarified this as a footnote in the Tables.

Reviewer #2

Comment 32: The "low" education group is an increasingly selected group over time, particularly over the last 30 years. The type of individual with less than an HS degree today is very different than in the 1990s, and is likely worse off on many dimensions. (My guess is that the fraction of the sample reporting "low" education has decreased over the study period considerably). Novosad and colleagues (AEJ Applied: https://www.aeaweb.org/articles?id=10.1257/app.20190297) consider this form of selection, and focus instead on percentiles of years of education (which are constant over time), using a partial identification approach to bound estimates. The Novosad paper suggests that the present estimates of increasing mortality in the low education group are almost certainly too large. I would love to see the selection corrected versions as a robustness check, at least.

Response 32: See Response 4 that addresses how the lung-cancer indexed method addresses period and cohort effects. Note that when we estimate smoking-attributable mortality, we used the risk ratios from the USSGR. The risk ratios actually incorporated cohort effects across three time periods (1959-1965, 1982-1988, and 2000-2010).[17] Furthermore, there has been literature that used quartiles with the same data source as ours to study mortality trends by gender, race and education[1] and their results on education are similar to ours. We have added some text on the mortality changes and education cohorts in the US, as discussed in Novosad (2022)[15].

Comment 33: Keeping #1 in mind, for the high education group, who not separate into "some college" (13-15) and four-year college and above (16+)? The latter group would be in direct conversation with work by Case, Deaton, Schwandt, Currie, Hayward and many others.

Response 33: See Response 3.

Comment 34: The counterfactual standard here is the "high education" group. However, mortality has slowed in this group, too. I wonder if an international counterfactual might be better for this exercise (e.g., https://pubmed.ncbi.nlm.nih.gov/37303714/). E Table 6 addresses this to some extent, but other data suggest that even the best off middle-aged Americans do worse than the poorest Europeans (https://www.nejm.org/doi/abs/10.1056/NEJMsa2408259).

Response 34: We added contextual comparison to OECD data, citing the relevant NEJM study. However, we believe that retaining a domestic comparison group is far more appropriate, and far more likely to influence debate within the US.

Comment 35: The authors acknowledge that smoking rates have gone down for lower educated Americans, just less so than other groups. That could explain diverging rates by education. But how does that square with an *increase* in mortality in this group? I might be misunderstanding, so apologies if so, but the only way this could occur is if smoking is deadlier today than it was previously I.e., we have P(smoking)*P(mortality|smoking), we know P(smoking) is going down, so P(mortality|smoking) must be going up. Has that been shown?

Response 35: We clarify that although smoking prevalence declined, cessation rates and cohort lag effects mean mortality attributable to past smoking remained high in specific sub-groups, most notably that smoking cessation in adults over age 50 in low education/SES groups remains low. As noted in response 20, the benefits of cessation may differ across education groups. We were surprised ourselves to see the rising lung cancer mortality in the lowest education groups, but these findings appear to be real and not an artifact of changes in classification of lung cancer deaths or in the denominator. Indeed, as pointed out in response 10, the misreporting of education on death certificates may underestimate lung cancer and other deaths in the lowest education group (but not likely in the combination of the two lowest education groups). We also add additional explanations (e.g., opioids, under-treated comorbidities) in quantifying their contribution to the excess deaths in low education groups.

Comment 36: There may be other methods to estimate smoking attributable fractions besides Doll and colleagues, e.g.: https://pmc.ncbi.nlm.nih.gov/articles/PMC6219821/ or https://pmc.ncbi.nlm.nih.gov/articles/PMC6351587/. It would be interesting to assess the robustness of the findings to alternate methods.

Response 36: We tested two alternative methods (e.g., Ezzati et al and Preston et al)[18,19] and show that the Peto method yields more stable and conservative estimates of smoking attributable mortality in nearly every educational/sex strata (Extended Figures 6-9).

Reviewer #3

Comment 37: The educational gradient in mortality is well established; so are gaps in specific causes of death (Miech et al. 2011), the contribution of smoking to the overall mortality gap (Ho & Fenelon 2015), and the contribution of education-based inequalities to excess deaths or life years lost (Sasson & Hayward 2019); critique of the "deaths of despair" hypotheses is also fairly old by now (Masters et al. 2017; Ruhm 2022). Thus, the study's key contribution is unclear with respect to the extant literature. Regrettably, the authors do not engage with much of this literature and do not attempt to highlight what is new about their analysis, findings, or conclusions.

Response 37: We politely disagree on the novel contributions of this paper. Please see response 1 and response 24. The hypotheses that smoking accounts for much of the stagnation in US survival is clearly stated in the title and in the introduction.

Comment 38: Responses to the previous reviewers were not particularly effective in answering key concerns about the methods or data. Missing data are poorly handled. Education misreporting in unlinked data is also a reason for concern, though I do not believe that it completely invalidates the findings. Unfortunately, the authors opted not to engage with these issues directly (e.g. conduct sensitivity analyses, benchmark against other sources) and merely acknowledged them in the limitations. For example, they reply that "mortality-linked surveys do not span nearly as long as three decades, we opted to use the unlinked data for this analysis." However, the NHIS linked mortality files date back the 1980s and permit precisely this type of analysis, at the very least for all-cause mortality. The estimates could also be compared with other surveys in later periods.

Response 39: Again, we would politely disagree. The earlier revision did address all the points raised by the previous reviewers. We explain the need not just to examine all-cause mortality but specific diseases. As noted in Response 11, the NHIS is simply too small to reliably quantify the observed relationships, and use of national mortality data by education group is preferred. We have further removed the early years of data (1989-91) which had a higher proportion of missing education data.

Comment 39: The third paragraph of the introduction states that "a proper understanding of the stagnation in US adult survival requires consideration of three key features of American society," namely racial and ethnic disparities in mortality, the prominence of smoking behavior in driving mortality patterns and trends, and inequalities in access to healthcare. While all of those factors are indeed important, no evidence or reasoning is given to support the claim that they should be singled out from many other demographic, behavioral, or socio-political factors. Furthermore, the paper goes on to examine only one of those factors (smoking). If racial disparities are so important for understanding the US mortality landscape, why does the study ignore them altogether and focuses only on non-Hispanic whites? Health insurance coverage is also completely absent from the data and analysis, so why mention it so prominently in the introduction?

Response 39: See Responses 1-4 above. We have re-written for clarity.

Comment 40: Prior research has shown that the most significant educational cutoff is having a college degree (Case & Deaton 2021; Sasson & Hayward 2019). However, the present study categorized educational attainment as less than high-school (<11 years of schooling), high-school (12 years), and higher (13+ years). What was the main reasoning behind this categorization?

Response 40: See response 3. In addition, we used 13+ years as the high education cutoff because our data points come from people who passed away in the last 30 years and whose age of death is between 30 to 79, which means that when they were seeking higher education many decades ago, having a bachelor's degree wasn't as ubiquitous as it is now. Thus, having 13+ or 16+ years of education wasn't likely to change our story. We added robustness checks with BA cutoff and the results were similar and show these in the appendix The use of 13+ years yields more conservative estimates of the absolute excess of deaths in the lowest education groups.

Comment 41: The paper includes a number of inaccuracies and odd phrasings. For example, it is claimed that US mortality started stagnating around 1980. That is simply not true. Stagnation started around 2010, which is well documented and studied. Another example is "stagnating survival has significant political repercussions" (p.1 second paragraph). Given the rest of the paragraph, I believe the authors meant that stagnant mortality has social and political causes, rather than consequences.

Response 41: Thank you for pointing out this subtlety. The re-write is now silent on this point. Note that relative to other countries, US survival started to stagnate from the 1980s (Reply Figure 2 below) but that within the US, the flatlining of survival mostly occurred in the last decade.

Redacted

Reply Figure 2

Responses' References

1. Leive, A. A. & Ruhm, C. J. Education Gradients in Mortality Trends by Gender and Race.
2. Jemal, A. *et al.* Mortality from Leading Causes by Education and Race in the United States, 2001. *Am. J. Prev. Med.* **34**, 1-8.e7 (2008).
3. Montez, J. K., Hummer, R. A., Hayward, M. D., Woo, H. & Rogers, R. G. Trends in the Educational Gradient of U.S. Adult Mortality From 1986 Through 2006 by Race, Gender, and Age Group. *Res. Aging* **33**, 145–171 (2011).
4. National Research Council (US) Committee on National Statistics. Vital Statistics: Summary of a Workshop. in *Vital Statistics* (National Academies Press, Washington, D.C., 2009).
5. Foote, C. L., Meara, E., Skinner, J. S. & Stewart, L. R. Explaining the Widening Divides in US Midlife Mortality: Is There a Smoking Gun? *NBER Work. Pap. Ser.* https://ssrn.com/abstract=5922429 (2025).
6. Meza, R., Cao, P., Jeon, J., Warner, K. E. & Levy, D. T. Trends in US Adult Smoking Prevalence, 2011 to 2022. *JAMA Health Forum* **4**, e234213 (2023).
7. Nkosi, L., Odani, S. & Agaku, I. T. 20-Year Trends in Tobacco Sales and Self-Reported Tobacco Use in the United States, 2000–2020. *Prev. Chronic. Dis.* **19**, 210435 (2022).
8. Rostron, B. L., Boies, J. L. & Arias, E. *Education Reporting and Classification on Death Certificates in the United States*. (U.S. Dept. of Health and Human Services, Centers for Disease Control and Prevention, National Center for Health Statistics ; For sale by the U.S. G.P.O., Supt. of Docs., Hyattsville, Md., Washington, DC, 2010).
9. Hendi, A. S. Trends in Education-Specific Life Expectancy, Data Quality, and Shifting Education Distributions: A Note on Recent Research. *Demography* **54**, 1203–1213 (2017).
10. Jin, J. *et al.* Individual and community-level risk for COVID-19 mortality in the United States. *Nat. Med.* **27**, 264–269 (2021).

11. Chu, L. *et al.* Floods and cause-specific mortality in the United States applying a triply robust approach. *Nat. Commun.* **16**, 2853 (2025).
12. Anzalone, A. J. *et al.* Higher mortality following SARS-CoV-2 infection in rural versus urban dwellers persists for two years post-infection. *Nat. Commun.* **16**, 8933 (2025).
13. United States Surgeon General. The Health Consequences of Smoking -- 50 Years of progress: A Report of the Surgeon General: (510072014-001). https://doi.org/10.1037/e510072014-001 (2014).
14. Cho, E. R., Brill, I. K., Gram, I. T., Brown, P. E. & Jha, P. Smoking Cessation and Short- and Longer-Term Mortality. *NEJM Evid.* **3**, (2024).
15. Novosad, P., Rafkin, C. & Asher, S. Mortality Change among Less Educated Americans. *Am. Econ. J. Appl. Econ.* **14**, 1–34 (2022).
16. Branch, B. Differences in Uninsured Rates by Race and Ethnicity Persist Even Among Those With Higher Educational Attainment. *Census.gov* https://www.census.gov/library/stories/2023/03/education-and-racial-disparities-in-health-insurance-coverage.html (2023).
17. Thun, M. J. *et al.* 50-Year Trends in Smoking-Related Mortality in the United States. *N. Engl. J. Med.* **368**, 351–364 (2013).
18. Ezzati, M. & Lopez, A. D. Estimates of global mortality attributable to smoking in 2000. *The Lancet* **362**, 847–852 (2003).
19. Preston, S. H., Glei, D. A. & Wilmoth, J. R. A new method for estimating smoking-attributable mortality in high-income countries. *Int. J. Epidemiol.* **39**, 430–438 (2010).

Response to Editors and Reviewer

Editor's Comments and Responses in separate document

Reviewer #3

Comment 1: As another reviewer previously commented, the authors use multiple indicators of mortality (risks, rates, excess deaths) interchangeably throughout the manuscript. This practice not only makes the paper difficult to follow, but also potentially undermines some of its key arguments. For example, the authors note in the discussion that "smoking accounts for over three-fifths of the trends in educational inequalities in mortality in the US." It appears that this statement is based on Figure 5, which has no reference in the text, and portrays the absolute number of excess deaths attributed to smoking among low and middle-educated Americans. However, the absolute number of excess deaths also depends on the population composition, and not only on disparities in mortality rates. Thus, arguments and hypotheses should be stated more accurately throughout the paper and particularly whether they relate to the actual US population, some hypothetical construct (e.g., age-standardized or stationary population), or the risk of dying from an individual's standpoint.

Response 2: Thank you. We revised the methods section to explicitly address the calculation from death rates to estimated excess death. For each age group and sex, we estimated the average annual rate of reduction (AARR) separately for each education group, using 1992 and 1993 separately as base years. To estimate excess death, we projected hypothetical death rates using the high-education group's AARR applied to each low- and middle-education group's own baseline rates.

Comment 2: Compositional changes are largely ignored, despite being critical for understanding mortality inequalities (see van Raalte et al. 2025). It is also unclear how these concerns are addressed by the Peto method as the authors assert in their rebuttal. Related to this issue, it is not entirely clear if step 1 of the Peto method entails age standardization as the age intervals used are fairly large (e.g., 30-64), which could also introduce biases in the numerator and denominator across education groups.

van Raalte, A., Vierboom, Y., & Martikainen, P. (2025). Revisiting within-cohort compositional change to understand mortality inequalities. Vienna Yearbook of Population Research, 23(1), 43-57.

Response 3: We revised the Discussion to address compositional changes within cohorts. Note that the Peto method standardizes within 5-year age groups in each of the ranges of 30-64 and 65-79, not for the whole age range.

Comment 3: Figures 3 & 4 do not seem to add up. For example, among men aged 30-64 the mortality rate attributed to smoking has more than doubled from 300 to 700 deaths per 100,000 per year (Figure 3). However, the breakdown to major smoking-attributable causes in Figure 4 scarcely add up to this total. What is missing?

Response 4: We have re-checked Figure 2 and Figures 3 and 4 for each sex. Figure 3 and 4 features smoking-attributable causes from a set of major causes for males and females, respectively, not all smoking-attributable mortality by causes. Our footnote specifies that not all smoking-attributable causes are shown.

Comment 4: Missing data: the authors excluded 1.5 million deaths with unknown educational attainment level. Although it is a relatively small proportion of all deaths observed during the study period (33 million), they may be concentrated in specific years and education groups. These cases should be treated with greater care.

Response 5: The 1.5 million deaths with missing education are indeed concentrated in the first couple of years after death certificated started to include education data in 1989. This is why we choose to estimate excess deaths beginning 1992.

Comment 5: It would help to include a detailed table with descriptive statistics showing the number of deaths in each education group/year.

Response 6: We think this would be duplicative of available data sources. We have mentioned in the data sharing that the code to generate these data are available in Code Ocean.

Comment 6: No code was provided.

Response 7: Updated code are now available through Code Ocean.
